# Theoretical analysis for the fluctuation in the electric parameters of the electroporated cells before and during the electrofusion

**DOI:** 10.1007/s11517-022-02683-0

**Published:** 2022-10-19

**Authors:** Sameh Sherif, Yehya H. Ghallab, Yehea Ismail

**Affiliations:** 1 Biomedical Engineering Department, Helwan University, Cairo, Egypt; 2 Center of Nanoelectronics and Devices (CND), Zewail City of Science and Technology, Giza, Egypt; 3 The American University in Cairo (AUC), Giza, Egypt

**Keywords:** Electroporation, Electrofusion, Ultra-shorted pulsed electric field, Transmembrane potential, Pore radius

## Abstract

An electric pulse with a sufficient amplitude can lead to electroporation of intracellular organelles. Also, the electric field can lead to electrofusion of the neighboring cells. In this paper, a finite element mathematical model was used to simulate the distribution, radius, and density of the pores. We simulated a mathematical model of the two neighbor cells to analyze the fluctuation in the electroporation parameters before the electrofusion under the ultra-shorted electric field pulse (i.e., impulse signal) for each cell separately and after the electrofusion under the ultra-shorted pulse. The analysis of the temporal and spatial distribution can lead to improving the mathematical models that are used to analyze both electroporation and electrofusion. The study combines the advantages of the nanosecond pulse to avoid the effect of the cell size on the electrofusion and the large-pore radius at the contact point between the cells.

**Graphical abstract:**

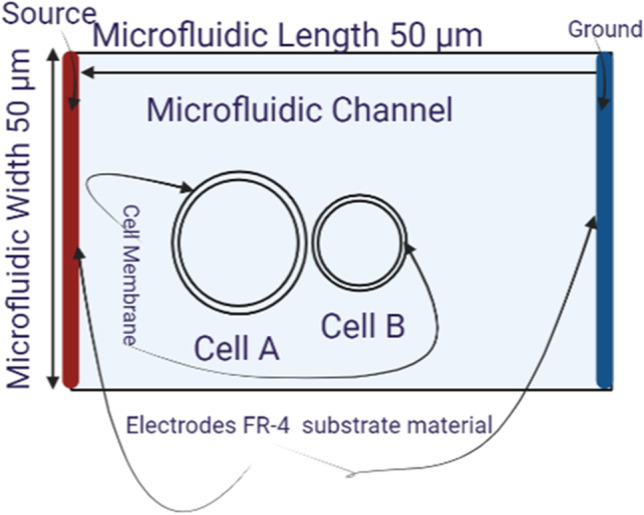

## Introduction

Electroporation is the reversible or irreversible permeabilization of the membrane of the cell under test in response to the application of electric fields across the membrane [1].

Electroporation is essentially a membrane electrical behavior that involves the response of lipid plasma membrane under the application of an external electric field [2].

When the influence of the applied electric field wears off, the cell membrane reverts to its impermeable state, but, in irreversible electroporation, the cell finally succumbs to the electroporation process and dies [3].

Electroporation [4], both reversible and irreversible, has become more important in medicine and biomedical technology [5], with applications spanning from cell ablation to gene transfection [6], nanomedicine, Clustered Regularly Interspaced Short Palindromic Repeats (CRISPR) manipulation [7], drug delivery [8], and various application [9]. It is found a lot of several studies focus on the drug delivery techniques as a vital factor in cell and tissue treatment [10, 11] or using PIPAC [12].

Single-cell micro-electroporation devices have been developed as a result of the development of micro/nano-electromechanical (MEMS) technology [1, 13, 14].

The two main phenomena of electroporation and electrofusion are related to achieving the electrical breakdown of the cell membrane [15].

The observation of membrane breakdown in electrically stimulated membranes has a strong effect on medical analysis [4].

For an electrical pulse [16, 17] with a specific duration, electroporation occurs passing through three stages: (1) charging the cell membrane, (2) creating the pores on the cell membrane, and (3) evolution of pore radii [18].

The highest pores [19] density occurs on two main poles (i.e., the depolarized and hyperpolarized poles); however, the largest pores are on the border of the electroporated regions of the cell [18].

The performance and efficiency of electroporation rely on different parameters. They are electric field parameters, biological parameters of exposure cells, and physical parameters, such as temperature [20, 21].

Electrofusion is one of the most efficient methods of cell fusion. It is used in many applications such as agriculture and the production of hybridomas [22].

Electroporation can be described by two main distributions (i.e., spatial and temporal distributions) [18]. Spatial and temporal distributions must be included in the electroporation study.

Temporal distribution is described by the analysis of the creation and the evaluation of the pores. These pores are obtained when the applied electric field strength is high and the membrane potential exceeds a specific threshold potential *V*th (∼ 0.2–1 V) [23], the permeabilization state occurs. The bilayer membrane will experience an electrical breakdown, the cell membrane becomes permeable and formed pores as a state in the electroporation path [24].

When the electric current penetrates the membrane, the cell membrane may be depolarized and/or hyperpolarized from its resting value (i.e., − 75 mV), which causes excitation or inhibition of the cell [25]. For this resting value, the cell needs up to 20 s to return to its original state (i.e., before the effect of the applied electric field) [26].

In this study, the density and radius of the pores are analyzed under the effect of an ultra-shorted electric field with and without the effect of neighbor cells. Also, the effect of neighbor cells on the electroporation parameters, such as membrane resealing fluctuation to achieve the electrofusion, has been studied and analyzed as well.

Different studies focused on the fluctuation in electroporation parameters based on the change of the applied electric field strength and the stimulus pulse parameters (i.e., shape and duration) [27]. Other studies represent the analysis of the fluctuation in the electrical parameters of the neighbor electroporated cells before the electrofusion [28, 29].

The basic mechanism of electroporation and electrofusion is a new and hot topic. Different studies in vivo and in vitro analysis to develop a model could simulate and explain the experimental observations [30–32].

In this study, we focus on the temporal process of creation and evolution of pores, and the spatial distribution of the transmembrane electric potential for B16-F1 and CHO cells.

The study included the effect of the ultra-shorted pulsed electric field with nanosecond (ns PEFS).

The previous studies of electrofusion depend on a microsecond pulsed electric field in the cell electrofusion treatment techniques [28].

It was difficult to achieve electrofusion for cells of different sizes. The reason for this problem is related to the effect of electroporation as a result of microsecond pulses that was greatly influenced by cell sizes. This study shows that the ability of ultra-shorted pulse in the range of nanosecond pulse (200 ns) can be used to avoid the difference between cell sizes to achieve electrofusion. Another main advantage for the selected electric pulse over the other previous studies is that pores induced by those short nanosecond pulses tended to be small (0.9 nm). But in this study with the different sizes B16-F1 and CHO cells for the tested cells, the pore radius was large enough (3.5 nm) and density was high (8.5 × ${10}^{14}{\mathrm{m}}^{-2}$) in the cells’ junction point.

The paper is organized as follows: Sect. 2 presents the definition of the mathematical model, the main geometry of the electrodes used to apply the electric field, different study cases, and mathematical equations that have been used in this study. Section 3 introduces the simulation results for different parameters for every single cell and the electrofusion response. Finally, the conclusion is drawn in Sect. 4.

## The mathematical model

### Mathematical model

**Fig. 1:**
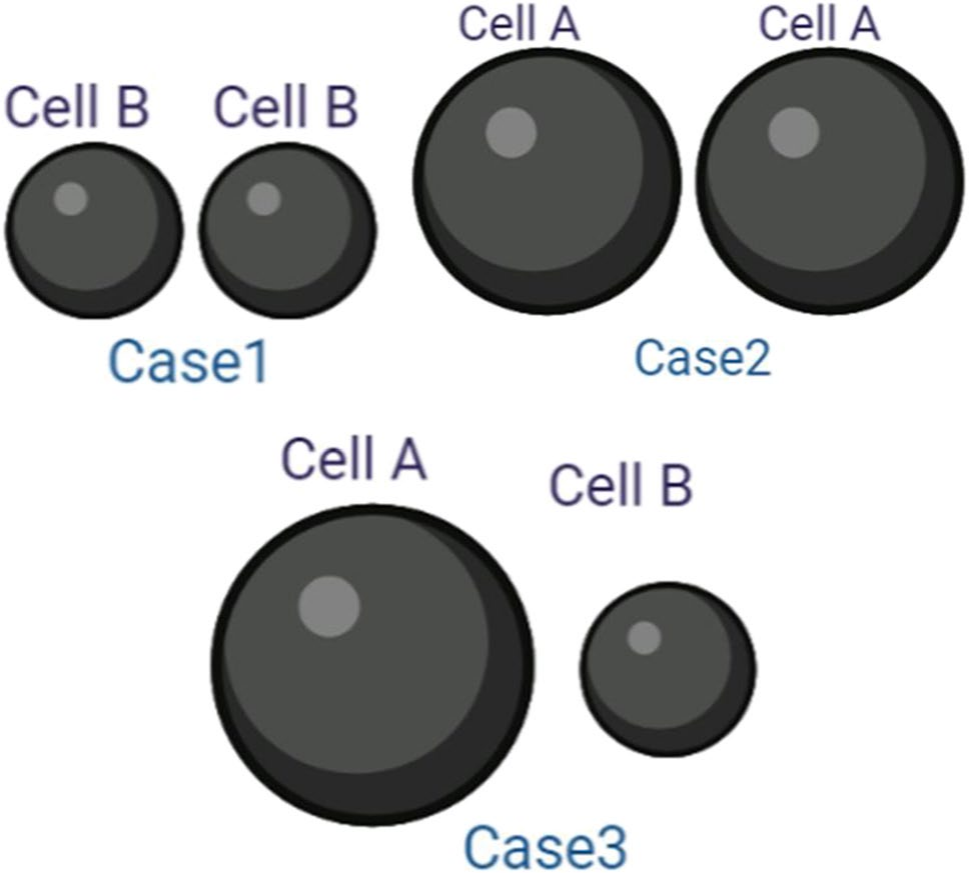
Schematic for the different cases used in this study. The cases include the study of the cells of the same size and different sizes B16-F1 and CHO cells and the fluctuation in the electroporation parameters due to the neighbor’s cells. Cell A is a large cell is B16-F1 cell, cell B is a CHO cell

As seen in Fig. 1, different cases used in this study and different considerations for the neighbor’s cells (B16-F1 and CHO cells). The three cases include the distribution as a function in the distance between the neighbor’s cells and the different sizes of each cell.

**Fig. 2:**
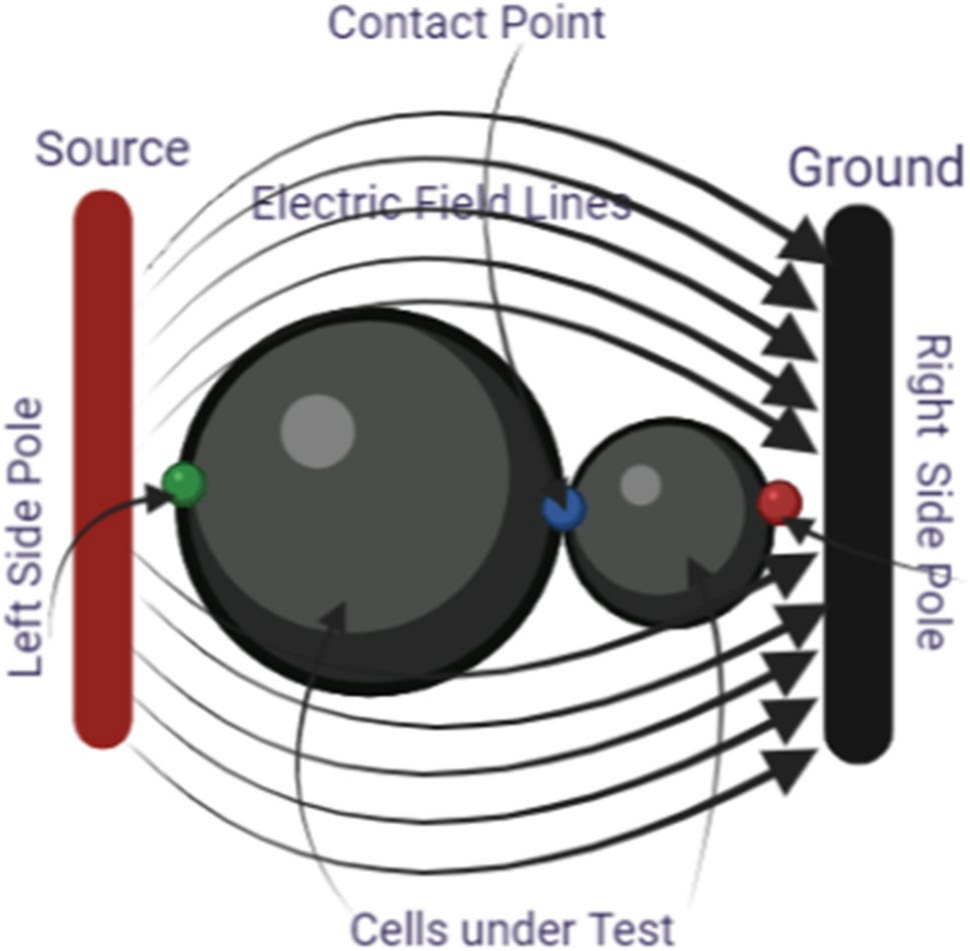
The schematic diagram of the main model used in this study with the definition for the main poles will be discussed in this study

Figure 2 represents the schematic diagram of the main model used in this study with the definition of the main poles that will be discussed in this study. The arrow shows the direction of the electric field from the high electric field terminal toward the lower electric field side. The study is divided into two parts. The first part shows the analysis of the electroporation parameters for every single cell, single-cell study to show the fluctuation in the cell membrane parameters at specific poles, such as hyperpolarized pole and depolarized pole as shown in Fig. 5. The second part shows the interaction between the cells at a specific point the left side pole and the right pole side which represents the border of the cells and the connection point which describes the contact area as described in Fig. 2.

**Fig. 3:**
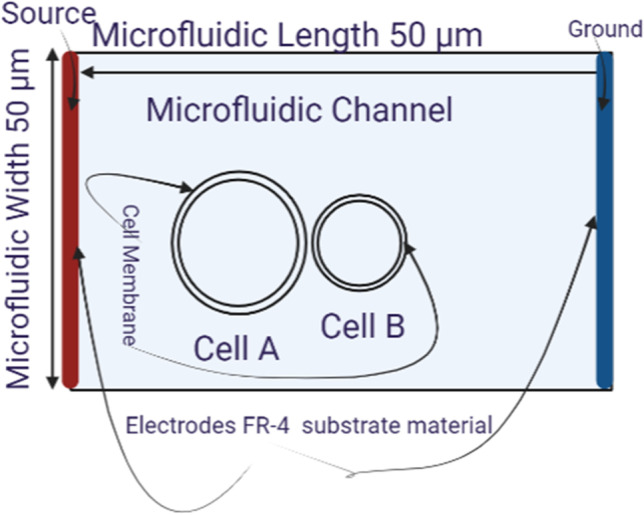
A schematic of the microfluidic channel was used to extract the main features of the electroporation

The system geometry consists of two parallel sensing electrodes, one of them represents the source and the other one represents the ground with a specific dimension. The electrode width is 50 μm and the distance between the electrodes is 50 μm as shown in Fig. 3. The copper was selected as electrode material and FR-4 is selected as the substrate material. The simulation uses phosphate buffer saline PBS as a medium surrounding the cell with electric conductivity 1 S/m and the permittivity is 80. The topology of the excitation on the right and left sides shows the influence of cells on each other away from the direct influence of the electric field. That means the contact point mainly affects be the communication of cells under the electric field and the neighbor cells.

The applied electric field range [1:1:4] kV/cm each value with the ultra-shorted pulsed electric field.

The excitation signal is a Heaviside function with a rising time of 10 ns and a duration of 200 ns. The Heaviside function can also be defined as the integral of the Dirac delta function $\delta (s)$ which given by $H\left(x\right)={\int }_{-\infty }^{x}\delta (s)ds$.

The effects of short pulses are considered non-thermal effects (avoid the thermally irreversible electroporation). When pulses are applied in the appropriate conditions, the increase in the temperature of the pulsed sample is negligible due to nanosecond pulse duration [28, 33]. When pulses are applied in the appropriate conditions, the increase in the temperature of the pulsed sample is negligible due to the short pulse duration.

The electric parameters of the exposure cells and other parameters used in this model are listed in Table 1.

**Table 1:** The simulation prameters

Parameters	Symbol	Value	Ref.
Cell (A) radius [B16-F1]	${R}_{a}$ (μm)	7.75	[30]
Cell (B) radius [CHO]	${R}_{b}$ (μm)	3.85	[30]
Extracellular medium conductivity	${\sigma }_{e}$ ($\mathrm{S}.{\mathrm{m}}^{-1}$)	1.2	[7]
Extracellular medium permittivity	${\varepsilon }_{e}$	72.28	[7]
Cytoplasmic conductivity	${\sigma }_{cp}$ ($\mathrm{S}.{\mathrm{m}}^{-1}$)	0.25	[7]
Cytoplasmic permittivity	${\varepsilon }_{cp}$	70	[7]
Cell membrane conductivity	${\sigma }_{cm}$ ($\mathrm{S}.{\mathrm{m}}^{-1}$)	5 · ${10}^{-7}$	[7]
Cell membrane permittivity	${\varepsilon }_{\mathrm{cm}}$	4.5	[7]
Cell membrane thickness	${d}_{m} (\mathrm{nm})$	5	[7]
Pore radius	${r}_{p} (\mathrm{nm})$	0.76	[7]
Electroporation constant	q	2.46	[7]
Electroporation parameter	$\propto ({\mathrm{m}}^{-2}.{\mathrm{s}}^{-1})$	109	[7]
A characteristic voltage of electroporation	${V}_{ep} (\mathrm{V})$	0.258	[7]
Minimum hydrophilic pore radius	${r}^{*}$ (nm)	0.51	[7]
Constant for pore radius evolution	${r}_{h}$ (nm)	0.97	[28]
Constant for pore radius evolution	${r}_{t}$ (nm)	0.31	[18]
Ionic diffusivity	${D}_{p}{\mathrm{m}}^{2}/\mathrm{S}$	1.6*${10}^{-9}$	[26]

### Mathematical equations

Electric potential V in the COMSOL 2D Model was extracted using the AC/DC module with time-dependent study solver using equation listed in [8].

1 $$-\nabla \left({\sigma }_{i}\nabla \mathrm{V}\right)-\nabla \partial \left({\varepsilon }_{i}\nabla \mathrm{V}\right)/\partial t=0$$

where ε and σ are the permittivity and conductivity, respectively. The index *i* for all subdomains such as the extracellular medium, liposomes cell, and nucleus. The plasma membrane was modeled as the boundary condition. According to [8], a cell with symmetric spherical configuration with RC components membrane exposed to an external electric field will polarize by transmembrane potentials ${V}_{m}$ occurs at each pole of the cell. The transmembrane potential can describe by

2 $${V}_{m}={~}^{3}\!\left/ \!{~}_{2}\right.*E*R*cos\theta$$

*E* represents the electric field strength, *R* is the radius of the cell, and *θ* is the angle between the direction of the electric field and the point vector on the membrane. The induced transmembrane voltage (ITV), determined as the difference between electric potentials on each side of the boundary, can be calculated by:

3 $$ITV={V}_{in}-{V}_{out}$$

The current density *J* through the shell membrane using distributed impedance boundary condition [8] Described by:

4 $$n.J={~}^{{\sigma }_{m}}\!\left/ \!{~}_{{d}_{m}}\right.\left(V-{V}_{ref}\right)+{~}^{{\varepsilon }_{m}}\!\left/ \!{~}_{{d}_{m}}\right.(\frac{\partial V}{\partial t}-\frac{\partial {V}_{ref}}{\partial t})$$

Here, *n* is the unit vector normal to the surface, *V* is inside potential, ${V}_{ref}$ is outside potential, ${\varepsilon }_{m}$ is the membrane permittivity, ${\sigma }_{m}$ is the membrane conductivity, and ${d}_{m}$ is the membrane thickness. To extract the membrane electroporation.

The pores formation is solved by a differential equation. The pores density number N accounts for the transmembrane potential values [8]:

5 $$\frac{\partial N}{\partial t}=\alpha .\mathit{exp}{\left({~}^{ITV}\!\left/ \!{~}_{{V}_{ep}}\right.\right)}^{2}.(1-\frac{N}{{N}_{0}}.\mathrm{exp}\left(-q{\left(\frac{ITV}{{V}_{ep}}\right)}^{2}\right))$$

where ${N}_{0}$ the membrane pores density when there is no transmembrane potential called a nonelectroporated membrane, and ${V}_{ep}, \alpha$, and $q$ are electroporation parameters.

The pore radius can calculate by the analysis for the following differential equation [15].

6 $$\frac{{dr}_{p}}{\partial t}=\frac{D}{{k}_{B}T}\left\{\frac{{V}_{m}^{2}{F}_{\mathrm{max}}}{1+{r}_{h}/\left({r}_{p}+{r}_{t}\right)}+4\beta {\left(\frac{{r}_{*}}{{r}_{p}}\right)}^{4}\frac{1}{{r}_{p}}-2\pi \gamma +2\pi {\sigma }_{eff}{r}_{p}+{F}_{elastic}\right\}$$

where ${D}_{p}$ the diffusion coefficients for the interactive transport. ${d}_{m}$ cell membrane thickness. ${r}_{p}$ pore radius.

The size of the pores is considered to be an important feature key in determining the efficacy of an electroporation treatment protocol. ${F}_{max}$ is the maximum electric force for ${V}_{m}=$ 1 V, ${r}_{h}$ and ${r}_{t}$ are constants, *β* is the steric repulsion energy, *r* ∗ is the minimum radius of hydrophilic pores, and *γ* is the energy per unit length of the pore’s edge.

## Simulation results for each single cell

The target is to extract the solution mathematical equations from [1:6] for the mathematical models, firstly we represent the solution for each cell separately. Then, the extraction for the electroporation’s parameters is based on the effect of neighboring effect.

**Fig. 4:**
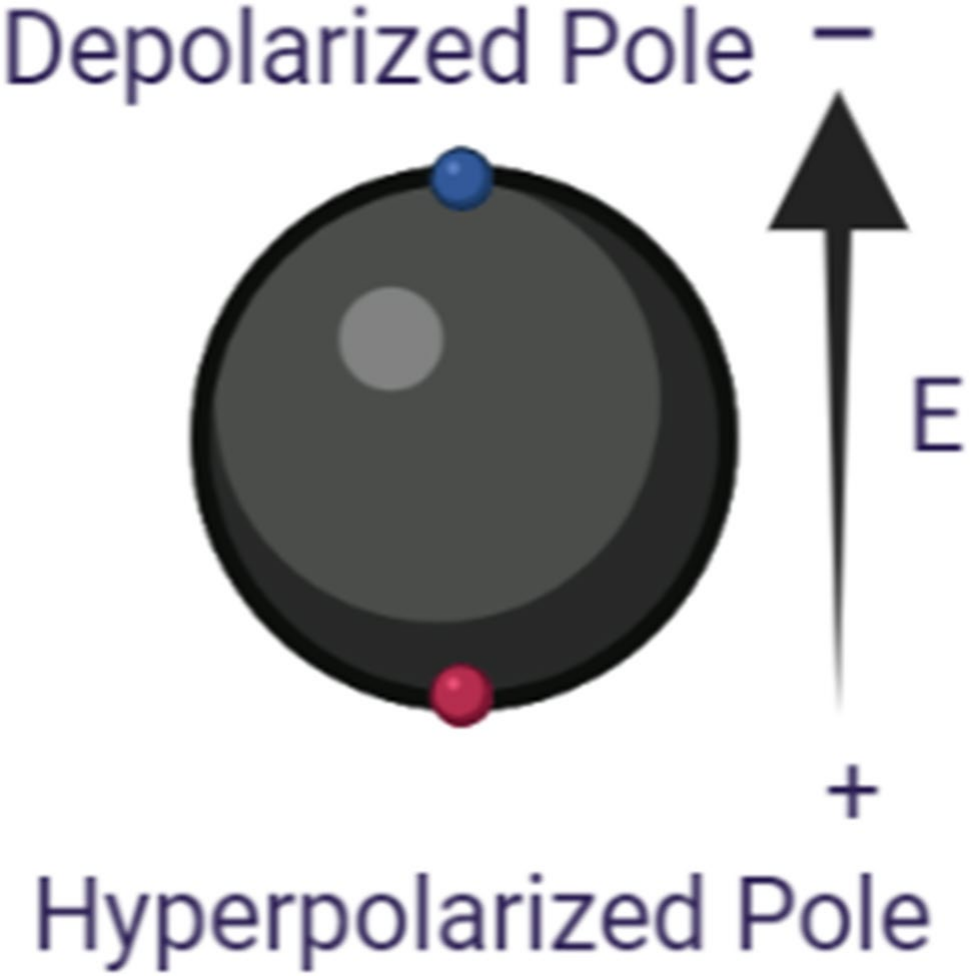
This figure schematic of a spherical cell considered in this study with the location of two main poles related to the applied electric field

Firstly, the analysis results are based on the extracted features at the two main poles: the hyperpolarized pole and depolarized pole as shown in Fig. 4.

### The electroporation parameters for the first small cell (cell B) as a single cell

**Fig. 5:**
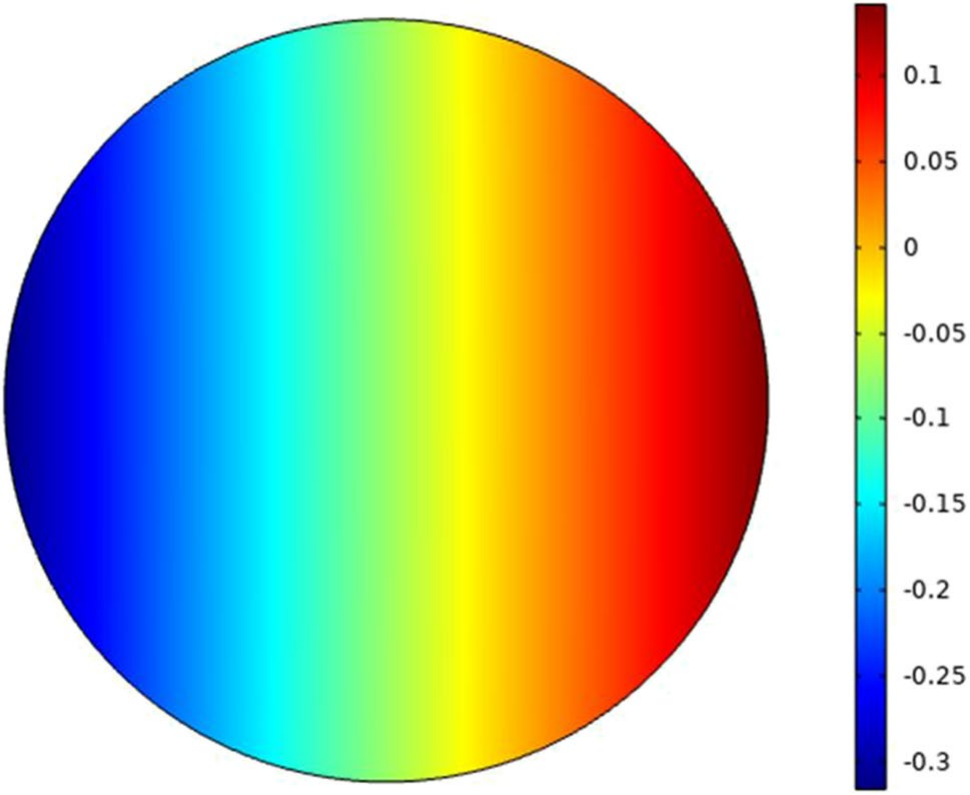
This figure shows the distribution of the internal voltage for cell B. The red region represents the internal voltage at the depolarized pole and the blue region represents the internal voltage at the hyperpolarized pole under the nanosecond pulse with the electric field strength 1 kV/cm and the represented figure at 300 ns after the end of the electric pulse

**Fig. 6:**
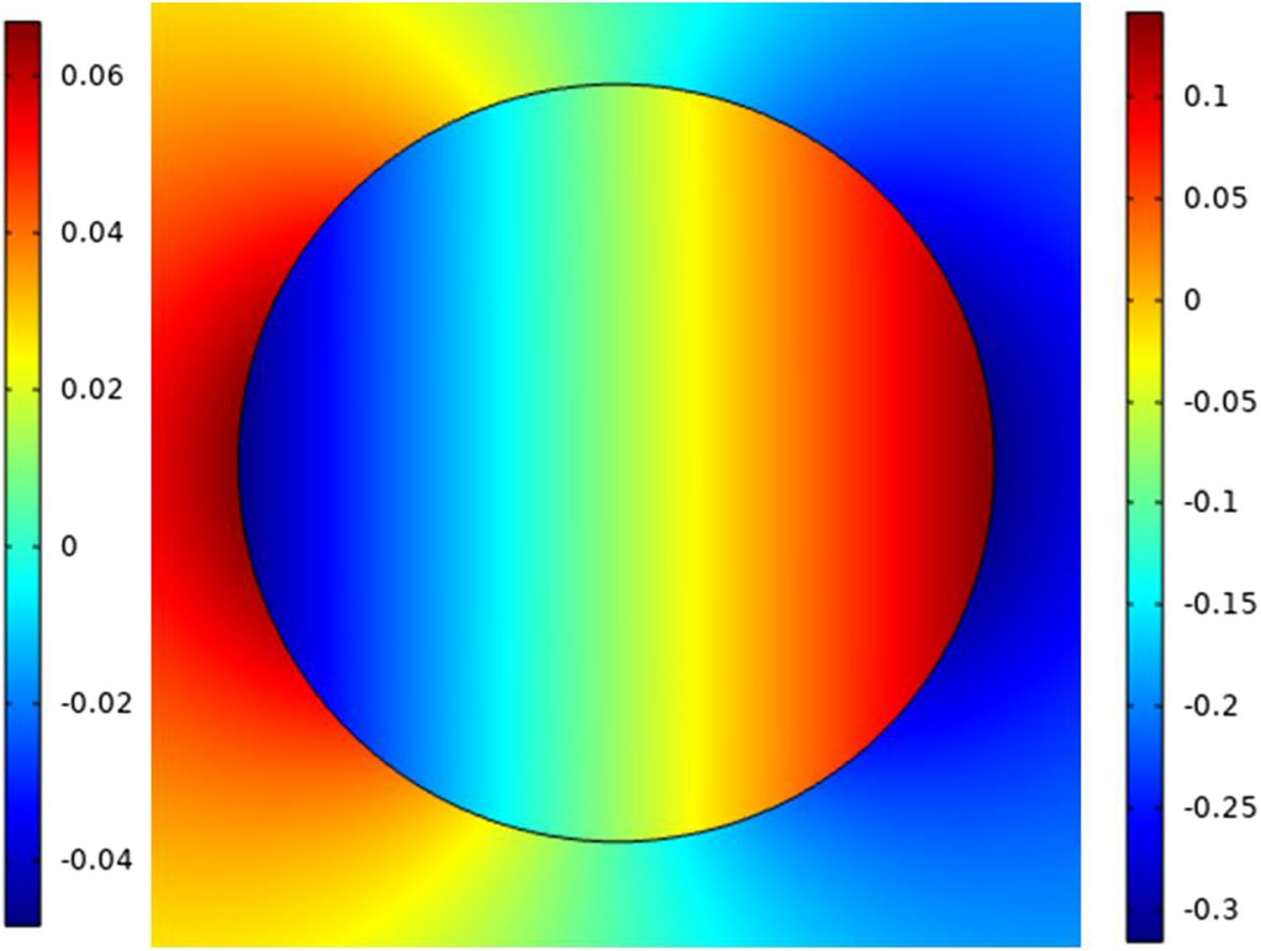
This figure shows the distribution of the electric field around the small cell related to the electric field inside the cell. The right indication legend for the electric field refers to the external electric field and the right one for the internal electric field

In this part the analysis for each cell as a single element without the effect of the neighbor cells to show the response of each cell separately under the selected excitation. The microfluidic system shown in Fig. 3 is used to extract the electroporation parameters for each cell and the different combinations as mentioned in Fig. 1. The internal voltage for the cell can be represented as the distribution in Fig. 5. The distribution consists of two main parts (i.e., the response of the hyperpolarized pole and the response of the depolarized pole). The blue color represents the hyperpolarizing anodic side, and the red color represents the depolarizing cathodic side at the end of the electric pulse 200 ns with the strength of the electric field 1 kV/cm. Figure 6 shows the distribution of the electric field around the cell related to the electric field inside the cell under the electric field strength 1 kV/cm and at the end of the pulse 200 ns.

### Pore radius for the first small cell (cell B)

**Fig. 7:**
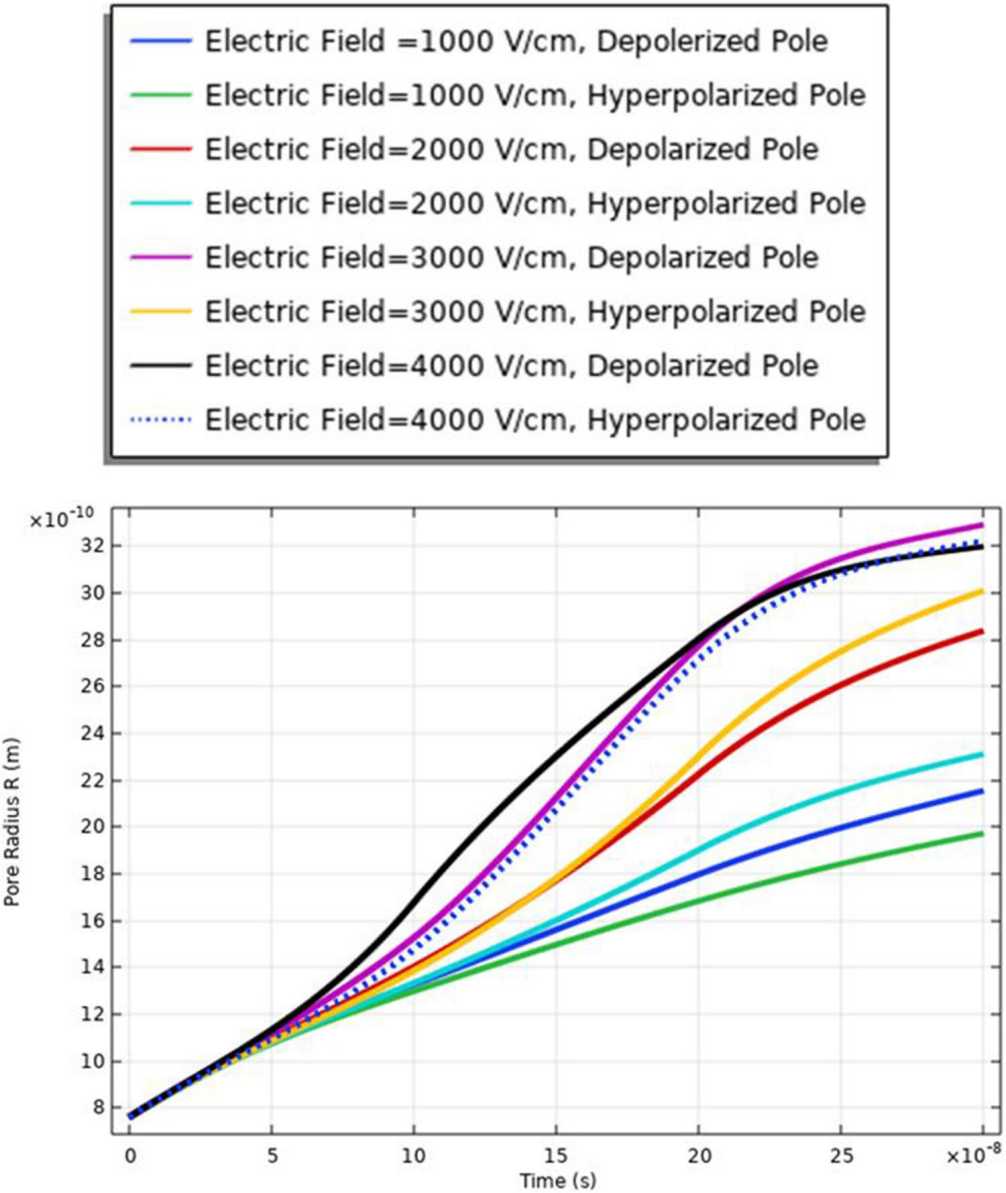
This figure shows the distribution of the pore radius at the selected point for each cell. The anodic side represents the hyperpolarized pole and the cathodic side represents the depolarized pole

The membrane response for the electroporation can translate into hydrophilic pores on the cell membrane surfaces. These pores permit the cell membrane to be a delivery system for ions, DNA, and different big molecules to pass through to the cell [16]. As mentioned in Table 1, small cell dimension cell B was used to show the effect of the selected electric field on different cells with different geometry. The analysis of the pore radius shows the effect of the selected excitation signal on the achievable pore radius. From Fig. 7, the maximum pore radius (3.3 ns) for the depolarized pole at the strength of the electric field equals 3 kV/cm. The increase in the pore radius is based on the increment in the electric field. The minimum pore radius is obtained at 1 kV/cm.

### Transmembrane potential (cell B)

In this part, the analysis of the transmembrane potential as a relation between the internal and external electric fields is represented in Eq. (3). The transmembrane potential shows the difference between the hyperpolarized pole and depolarized pole. Also, the analysis of the transmembrane potential shows the ability of the cell to achieve the critical value Vth, which as defined in [7] permeabilized state, when an appropriate external electric field is applied. When the induced membrane potential exceeds a threshold voltage, Vth (∼ 0.2–1 V), the permeabilization state occurs and the pores density and transmembrane potential can identify it.

**Fig. 8:**
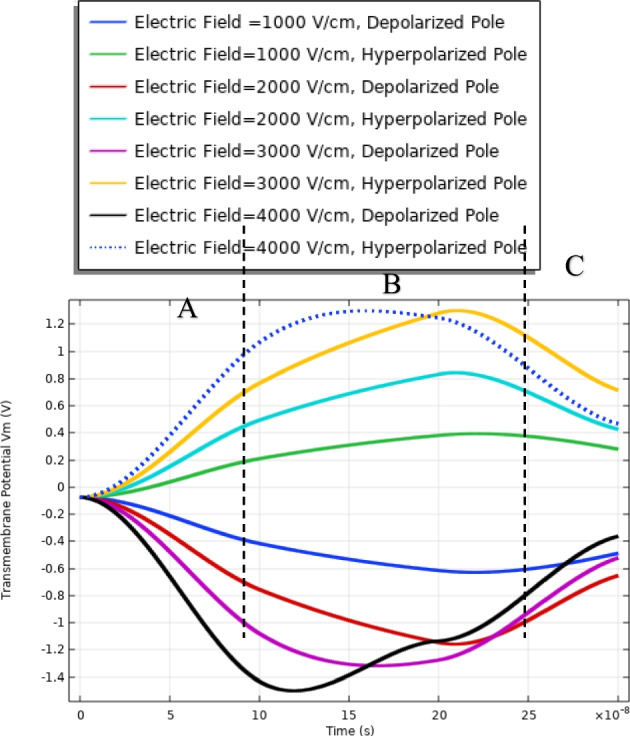
This figure shows the transmembrane potential for a small cell under the excitation electric pulse with duration 200 ns and the electric field distribution [1:1:4] kV/cm

The transmembrane distribution in Fig. 8 can be divided into three main regions of charging time A, B, and C. Region A is called the charging time. Note that the charging time as described in [18] decreases with the increase in membrane leakiness. The second region B is called the pore nucleation. The last region C is called the pore evaluation. The charging stage begins when the electric field is applied and ends with the creation of the first pore anywhere on the cell membrane. Pore nucleation starts when its transmembrane potential exceeds a threshold value of $\sim$ 1 V. Pore evolution is the slowest process than either pore nucleation or changes in the transmembrane potential.

### Pores density (cell B)

**Fig. 9:**
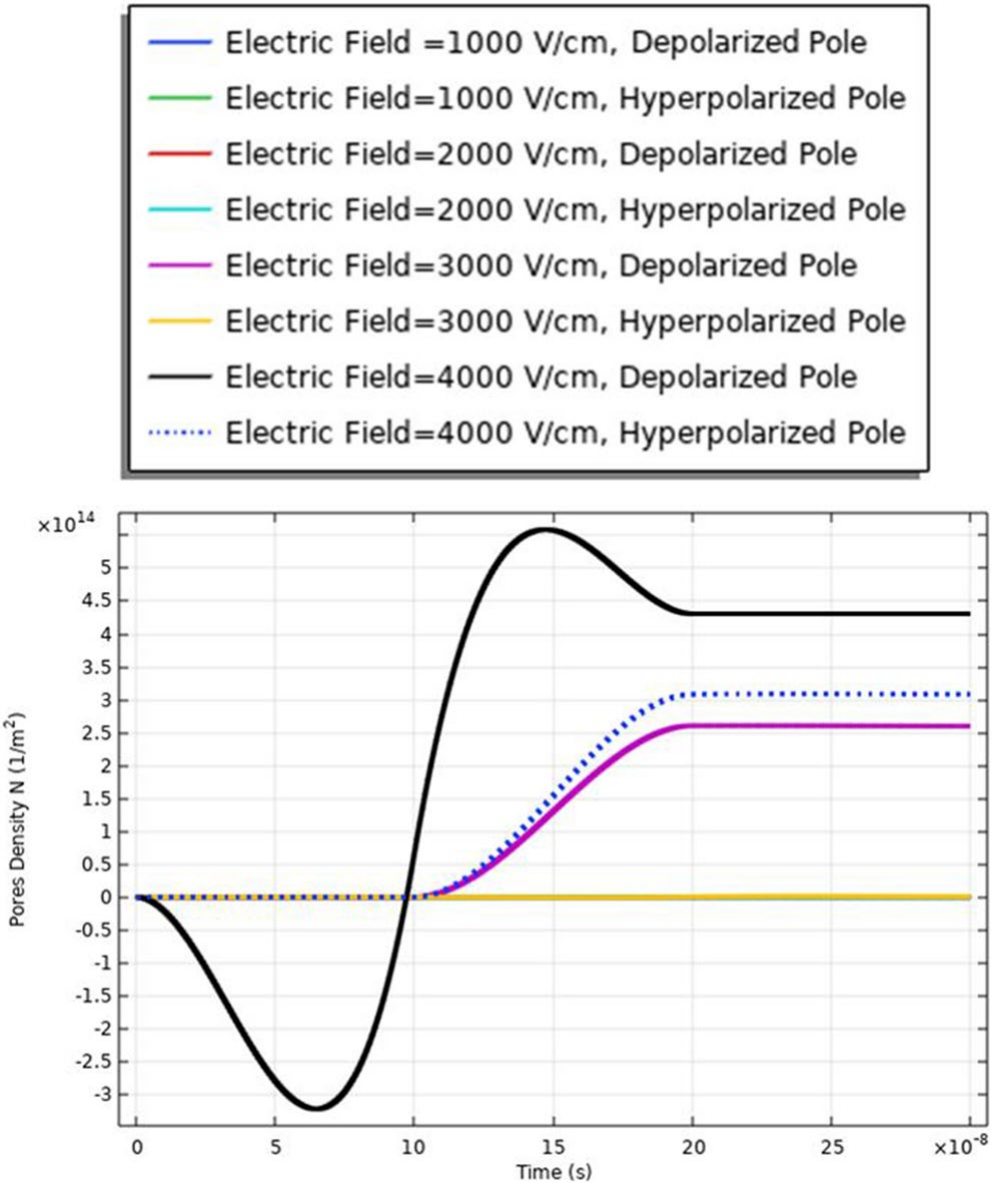
This figure shows the distribution of the density of the pores for cell B. The difference between the maximum values of the density of the pores at the depolarized pole and depolarized pole around 1.5*${10}^{14}{\mathrm{m}}^{-2}.$ The evaluation versus time axes [0:1 ns:300 ns]

To enhance the evaluation for the electroporation and the electrofusion under the applied electric field with nanopulse, the evaluations for the density of the pores for the small, selected pulse are represented in Fig. 9. The pores density extraction defines the combination between small pore’s radius and large-pore radius. The distribution of the density of the pores at specific poles as a function of the time is represented in Fig. 9. The maximum pore density at the depolarized pole at 4 kV/cm. The maximum value of the pore’s density equals (4.5*${10}^{14}{\mathrm{m}}^{-2}$). The gradual increase in density of the pores as the electric field gradually increases. The highest pores density occurs at the border of the electroporated region of the cell represented by the hyperpolarized pole and depolarized pole.

### The membrane resealing (cell B)

**Fig. 10:**
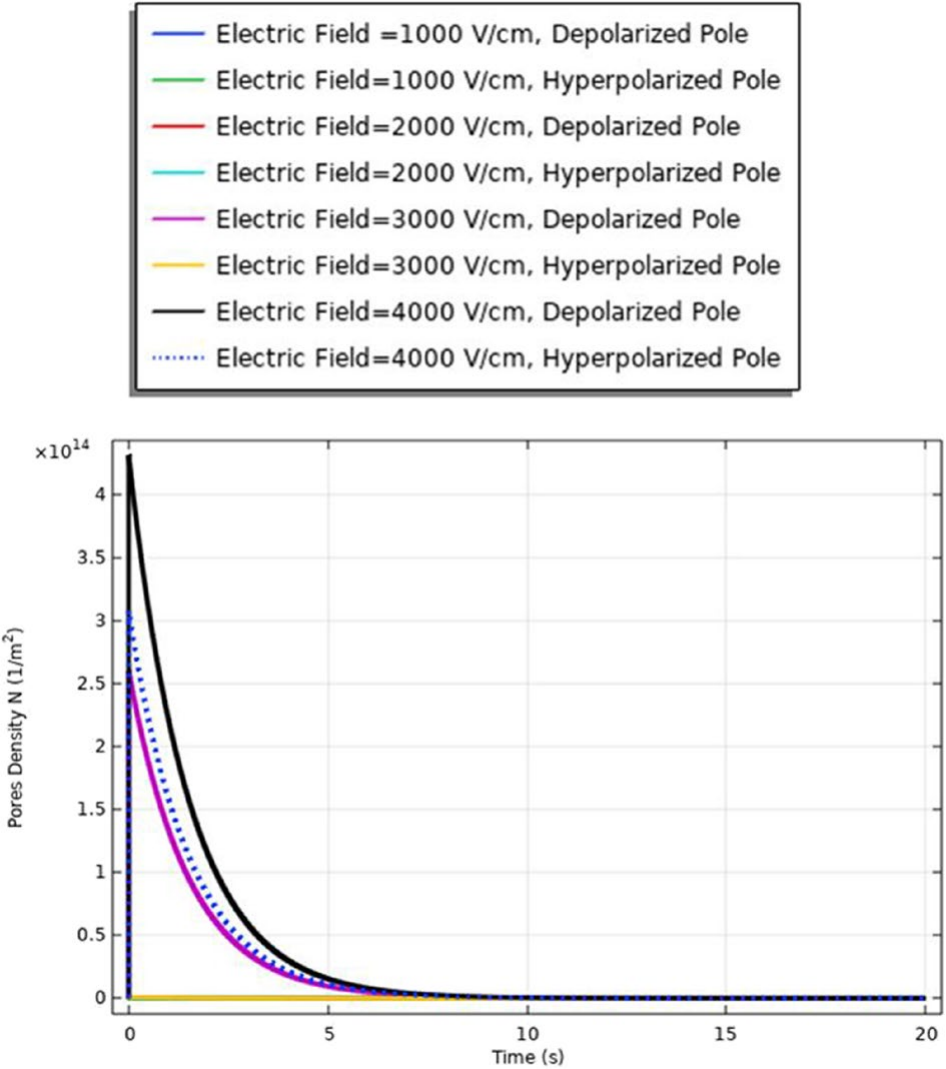
This figure shows the membrane resealing for the small cell (cell B radius = 3.85 μm) under electric stimulus pulse with nanosecond duration

The electroporation process has three main stages. They are the nonpermeabilized state, permeabilized state, and recovery state. (1) Nonpermeabilized state: the membrane before the external field is applied; (2) permeabilized state when an appropriate external electric field is applied. When the induced membrane potential exceeds a threshold voltage, Vth (∼ 0.2–1 V), the permeabilization state occurs and the pores density and transmembrane potential controls it. For cells, the necessary single electric field applied is in the range of ${10}^{3}$ − ${10}^{4}$ V/cm, the exact value depends on the cell size [17] to achieve the permeable state. (3) Recovery state refers to the gradually resealing of the cell membrane at the induced potential less than the irreversible breakdown. To evaluate the recovery state, the definition for the membrane resealing under the selected electric pulse is represented in Fig. 10. The achievable of the membrane resealing with the time under the selected electric pulse avoids the deformation of the cell membrane.

### Electroporation parameters for the second large cell (cell A)

**Fig. 11:**
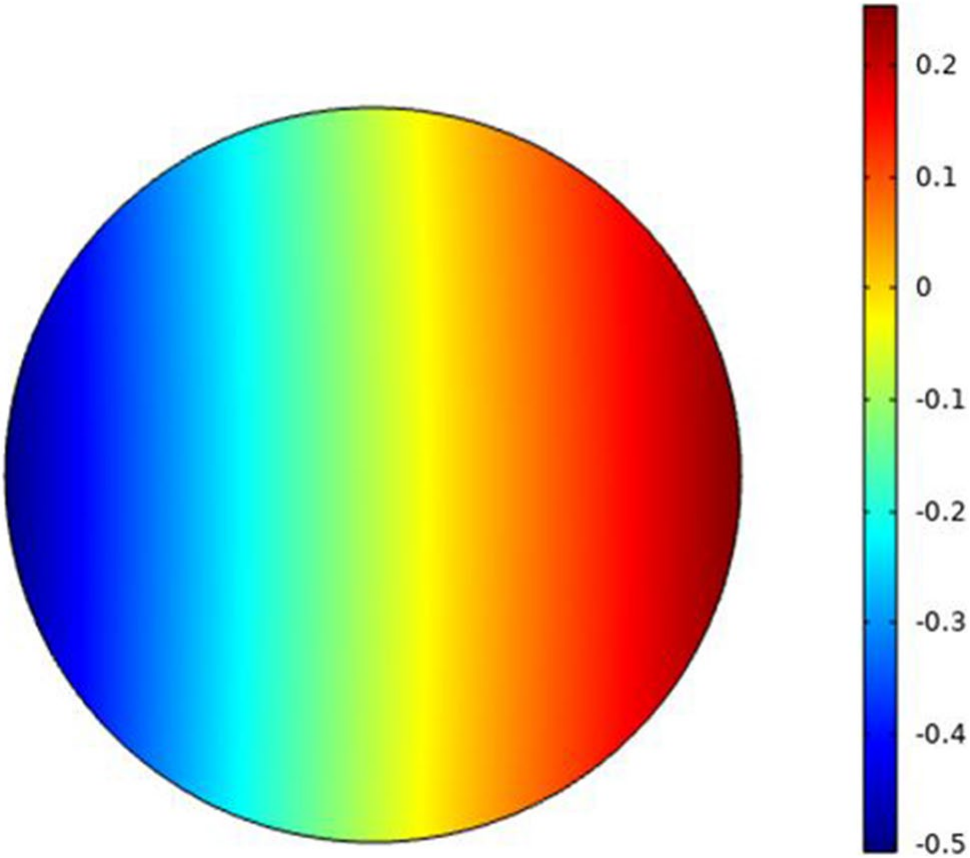
This figure shows the distribution of the internal voltage for the large cell-cell A. The red region represents the internal voltage at the depolarized pole and the blue region represents the internal voltage at the hyperpolarized pole under the nanosecond pulse with the electric field strength 1 kV/cm and the represented figure at 300 ns after the end of the electric pulse

The second step for the analysis is to study the effect of the electric pulse on the second cell that has a large radius as mentioned in Table 1. The difference between the internal voltage of different cells based on cell size can be extracted from Figs. 5 and 11.

**Fig. 12:**
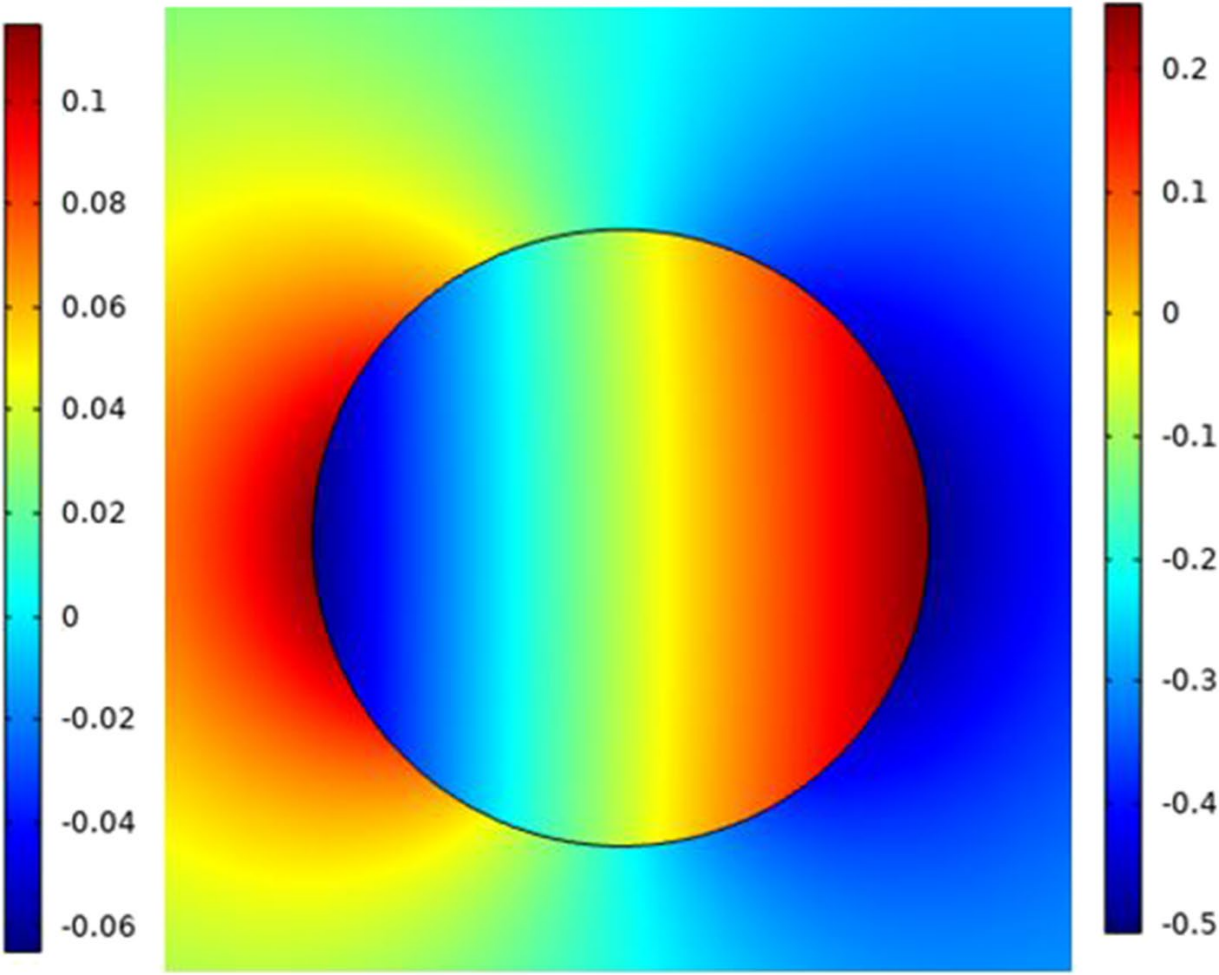
This figure shows the distribution of the electric field around the large cell related to the electric field inside the cell. The right indication legend for the electric field refers to the external electric field and the right one for the internal electric field. The distribution shows how can the electric field affected by the cell dimensions

The two main poles on the red side refer to depolarized pole and the blue side refers to the hyperpolarized pole. The distribution of the electric field around the cell and inside the cell can is shown in Fig. 12. Typical to cell B, we can define the same features for cell A.

**Fig. 13:**
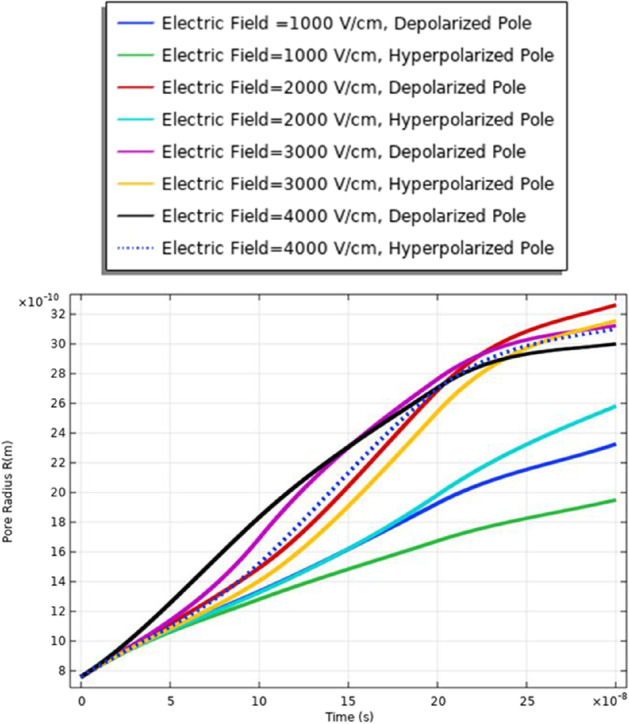
This figure shows the distribution of the pore radius for cell A at the selected point for each cell. The anodic side represents the hyperpolarized pole and the cathodic side represents the depolarized pole

Figure 13 and 16 used to define the electric parameters for cell A under the selected pulse. The difference between cells A and B can be represented by the data in Table 2. It is found different notifications between different cells under the same electric pulse. The difference between cells A and B in the pore radius feature can be noticed from Figs. 7 and 13; the difference between the hyperpolarized and depolarized pole at different strengths of the electric field is as follows: cell A: 31 ns at 4 kV/cm for the hyperpolarized pole and cell B: 32 ns at 4 kV/cm for the hyperpolarized pole.

**Table 2:** The simulation extracted features

Features	Cell A	Cell B
The max. pore radius range	3.3 nm at the electric field strength 2 kV/cm for the depolarized pole.	3.3 nm at the electric field strength 3 kV/cm for the depolarized pole.
The max. pores density	6*${10}^{14}/{\mathrm{m}}^{2}$	4.5*${10}^{14}/{\mathrm{m}}^{2}$
The charging time	8$*{10}^{-8} \mathrm{s}$ at the electric field strength 4 kV/cm	9$*{10}^{-8}$ s at the electric field strength 4 kV/cm

The delay between the two cells in the charging time concerning the transmembrane potential, a delay time of 1:1.5*${10}^{8}\mathrm{ s}$ between two cells at different strengths for the applied electric field. Cell A is charged faster than cell B at different electric field strengths. Also, the maximum value of the transmembrane potential is 1.4 V at the 3 kV/cm for cell A and 1.2 V at the same electric field strength for cell B.

**Fig. 14:**
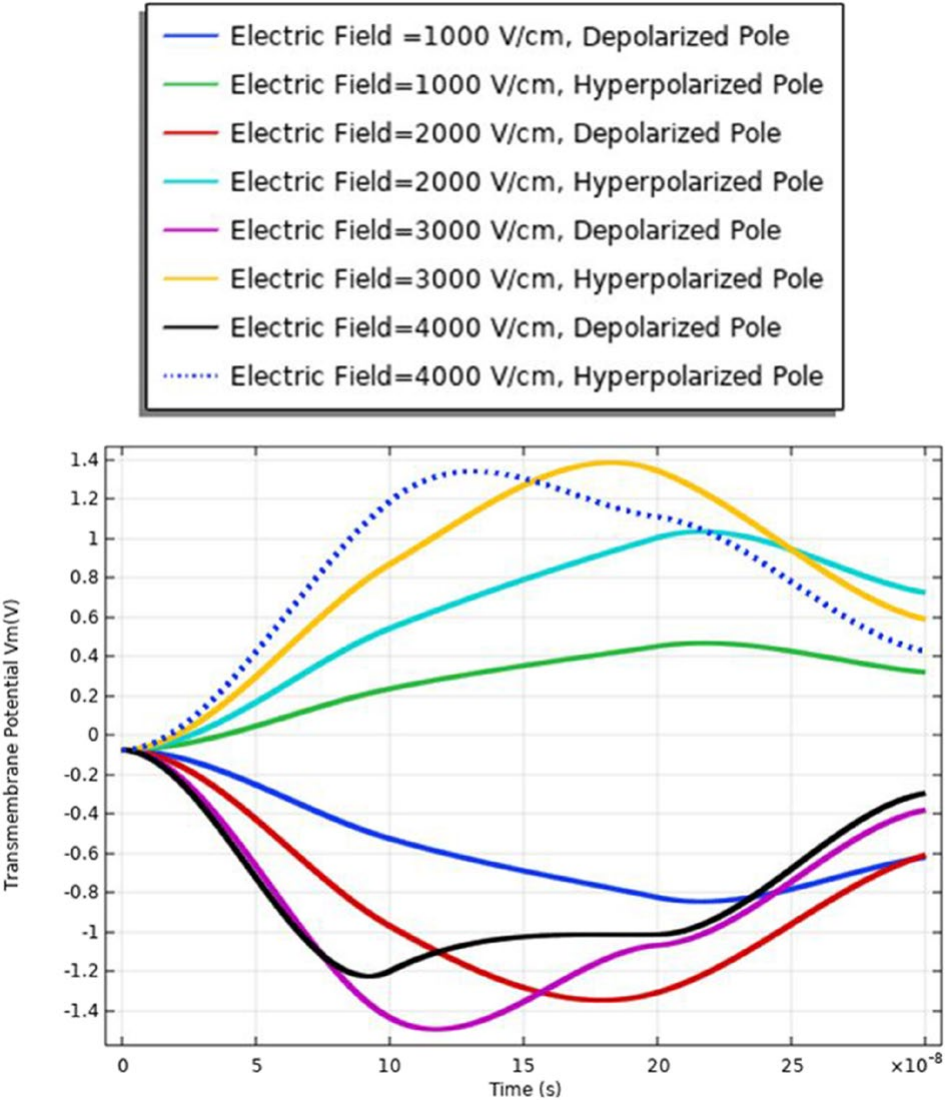
This figure shows the transmembrane potential for cell A under the excitation electric pulse with duration 200 ns and the electric field distribution [1:1:4] kV/cm

**Fig. 15:**
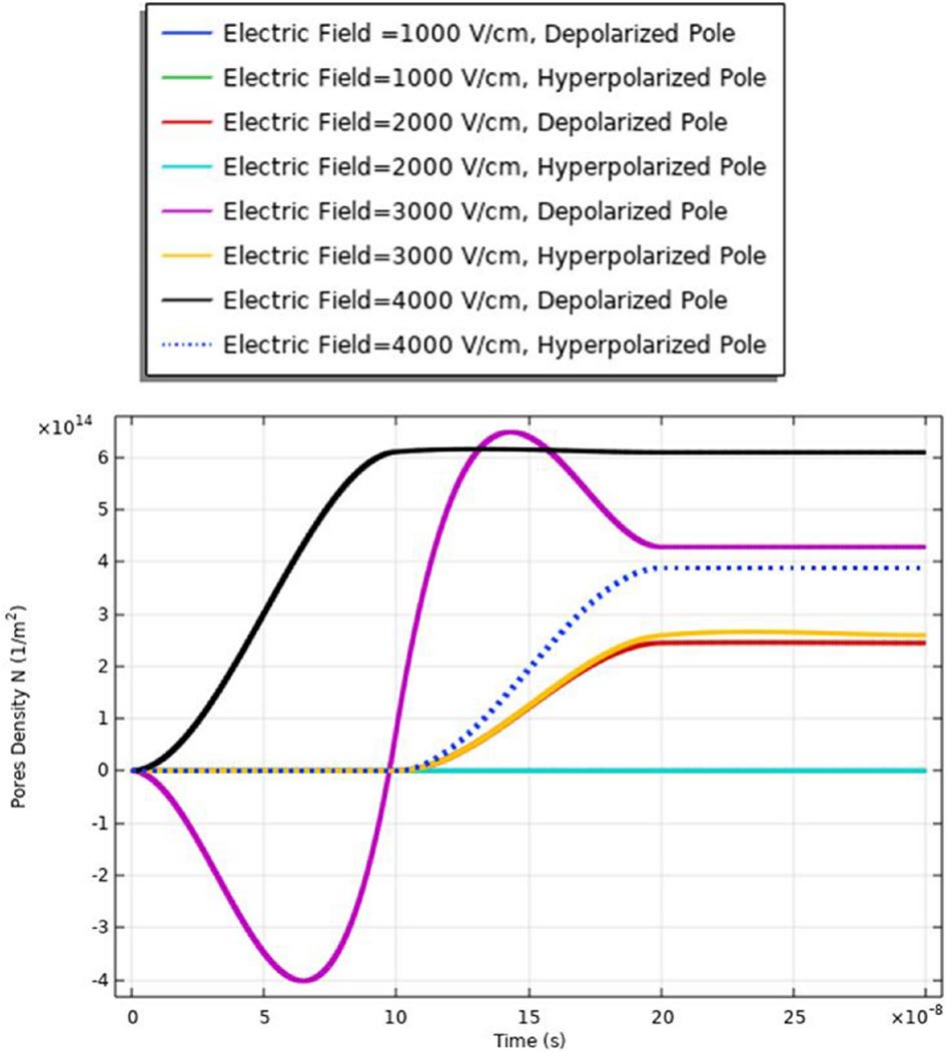
This figure shows the distribution of the density of the pores for cell A

**Fig. 16:**
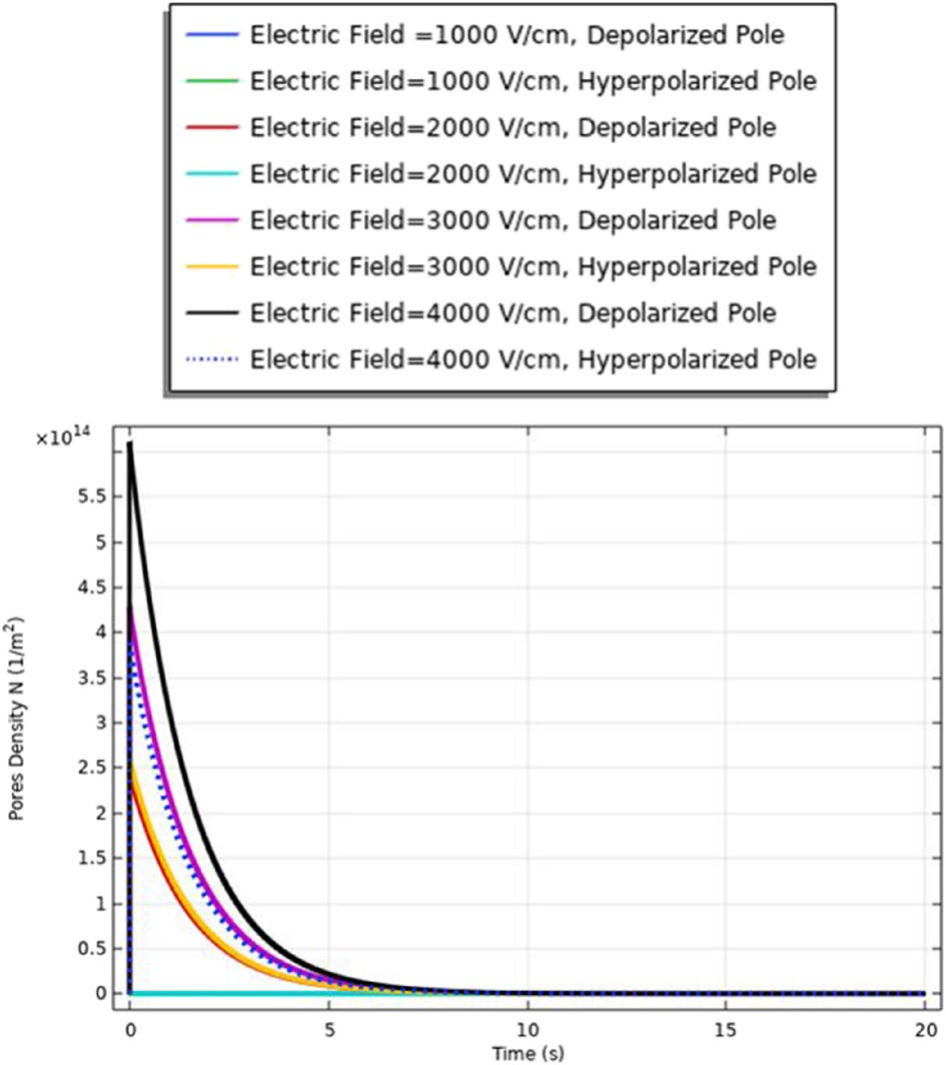
This figure shows the membrane resealing for the small cell (cell A radius = 7.75 μm) under electric stimulus pulse with nanosecond duration

The different responses based on the cell-specific parameters, from Eq. 2, found that the transmembrane potential is proportional to the cell radius. This relation between the cell radius and the transmembrane potential can describe the reasons for the difference between cell A and cell B's response under the same excitation signal. Another difference between the two cells from the distribution of the transmembrane potential Figs. 8 and 14 is the settling voltage, membrane resealing (Figs. 10 and 16), and the Pores density (Figs. 9 and 15) at different poles at the different electric field strengths.

### The electrofusion parameters for the neighboring cells

In this part, we represent the analysis for the different parameters such as the transmembrane potential, the pores density [34], and the pore radius based on the effect of the electrofusion [35] effect between the neighbor cells.

The fluctuation between the normal response of the cells under the same electric parameters and the electric pulse shown for every single cell separately without any effect from the neighbor cells has been discussed in paragraphs [3.1:3.6]

The next step is to study the features based on the effect of neighbor cells in cases 1, 2, and 3.

**Fig. 17:**
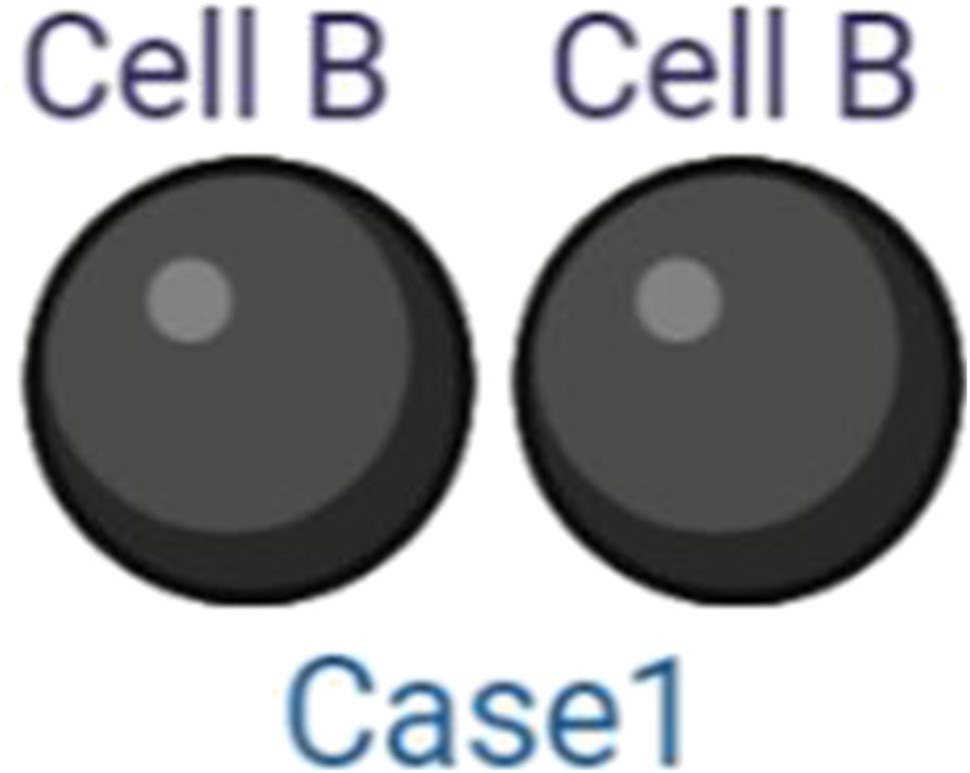
This figure shows the selected case with the need to define the effect of the selective pulse and the effectiveness of the treatment method on the same cell size under the electrofusion.

The first and second cases are based on the effect of the same cell sizes (Figs. 17 and 23) and the third case is for the different cells with different sizes (Fig. 28).

The Microfluidic device with predefined dimensions Fig. 3 was used to analyze the two neighbor’s cells Fig. 1 and the fluctuations in the electroporation and electrofusion based on the selected cases are discussed.

The strength of the selected treatment method can define by different definitions the ability of the selected treatment method to define the electrofusion for the different cells and the ability to differentiate between the contact point, the small cell, and the large cell.

**Fig. 18:**
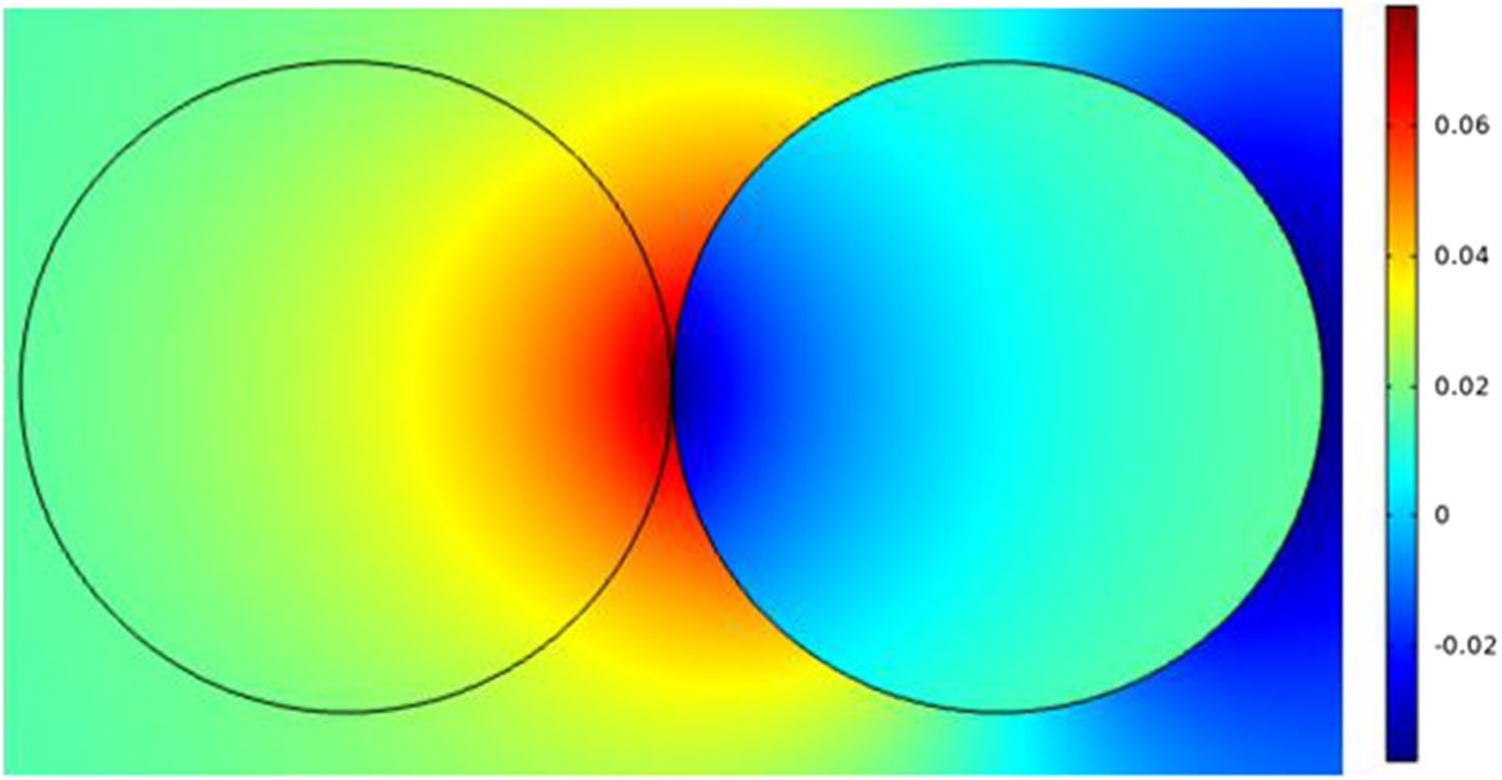
This figure shows the distribution of the electric field inside the cell and for the environment around the cell under the nanosecond pulse. The distribution at the end of the pulse and the smallest value of the electric field is 1 kV/cm. Note that as the electric field increase the distribution of the electric field also increases. The distribution also shows the high electric field at the contact region between the two cells

**Fig. 19:**
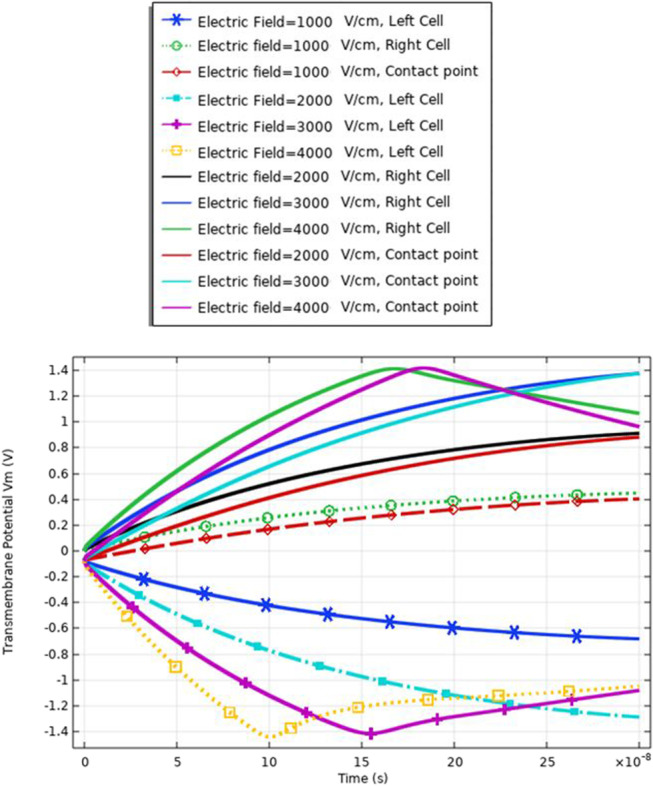
This figure shows the transmembrane potential under the nanosecond electric pulse with the distribution of the electric field [1:1:4] kV/cm. The response for the transmembrane potential is based on the same small size of the cell

**Fig. 20:**
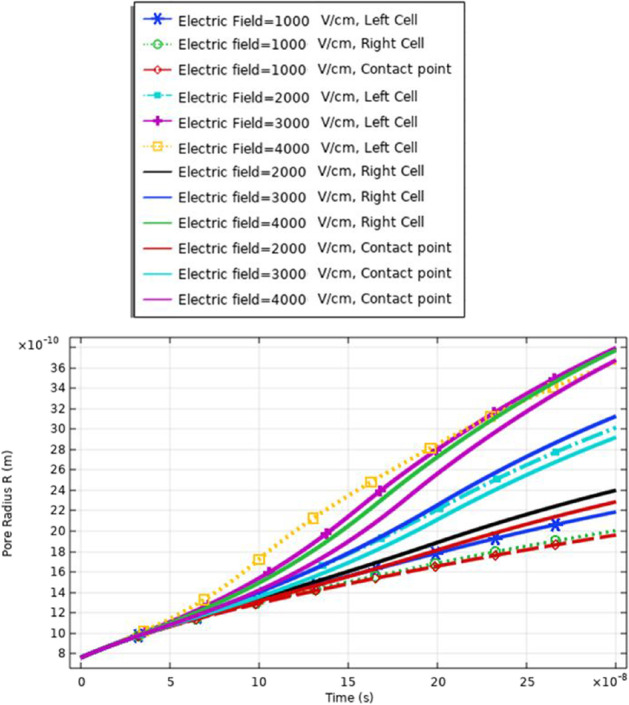
This figure shows the distribution pore radius at different selected points related to the left cell, the right cell, and the contact point between the two cells

**Fig. 21:**
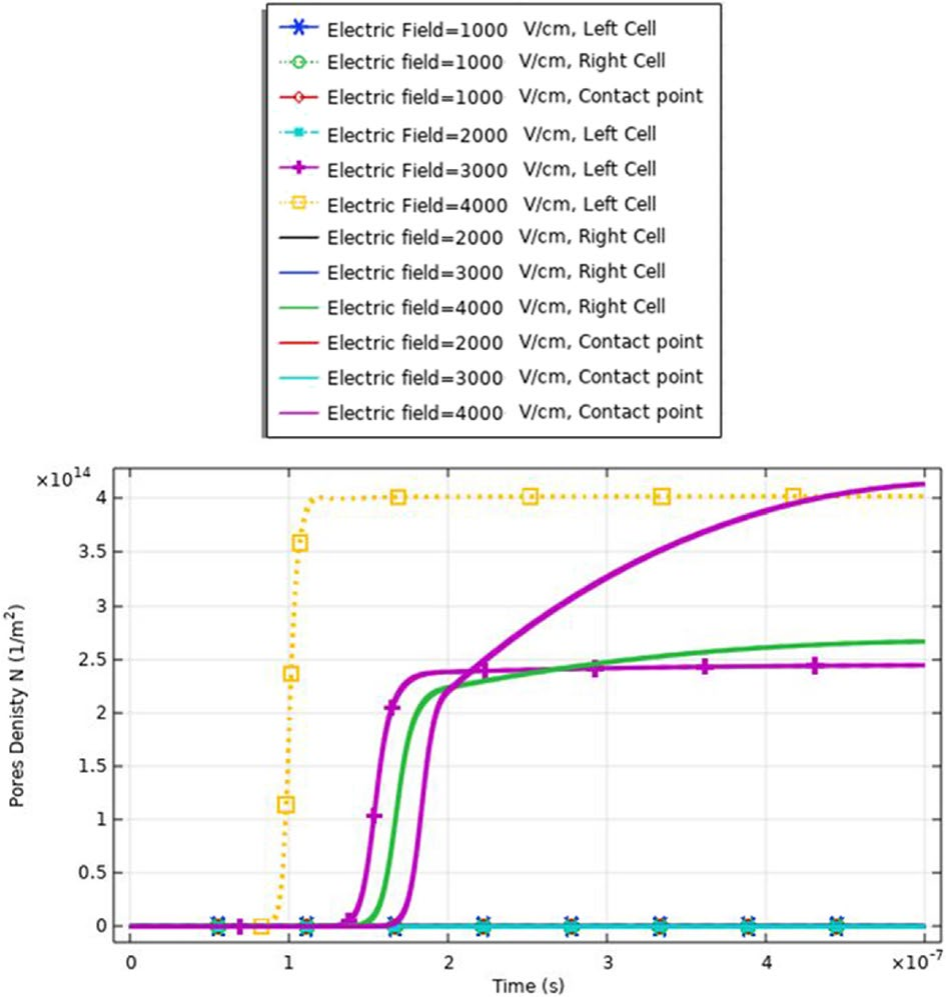
This figure shows the distribution of the density of the pores. The distribution for the same small cell size

**Fig. 22:**
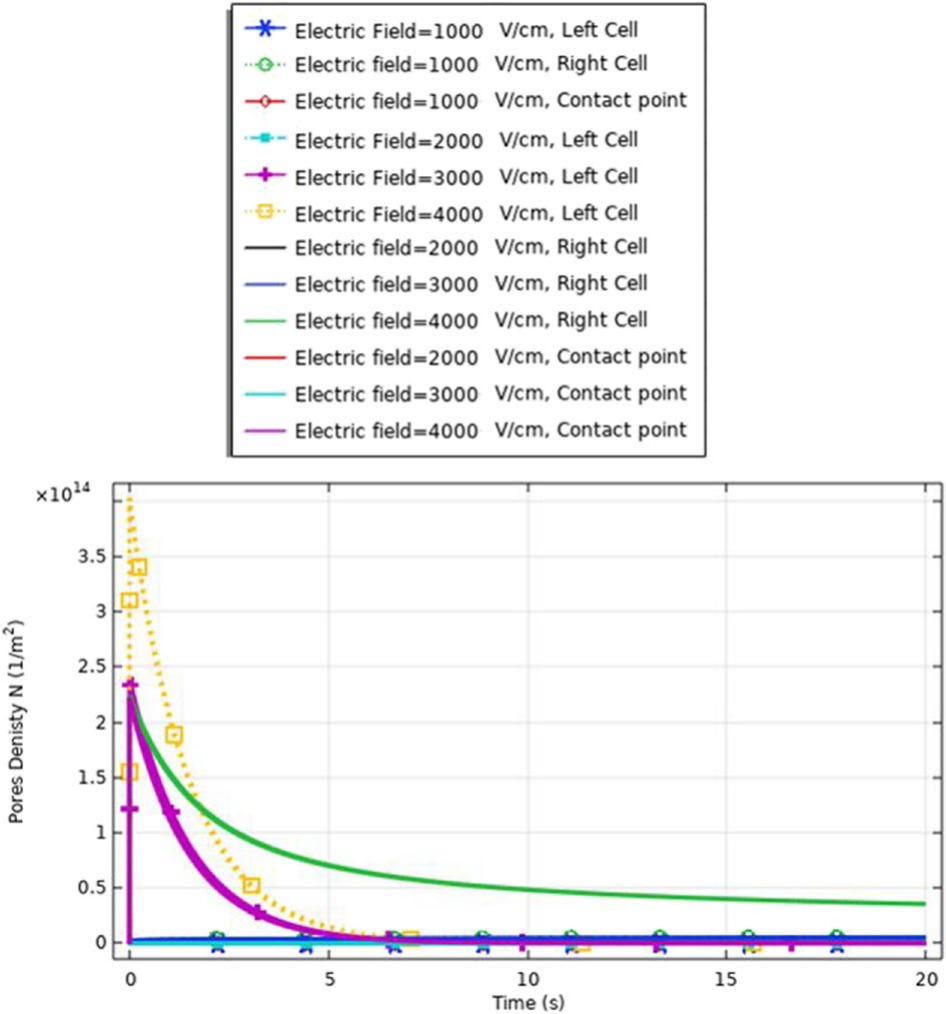
This figure shows the membrane resealing response for the same cell size with the different electric field strength. The membrane not completely resealing increases the probability of the electrofusion between the different cells in the presence of the same cell

The definition for these differences can be clear if we represent the different solutions for the different mathematical equations i.e. the pore radius (Figs. 20, 24 and 29), the transmembrane potential (Figs. 18, 19, 27 and 31), the density of the pores (Figs. 21, 25 and 30), and the membrane resealing (Figs. 22, 26 and 32). The ability to differ between the different points and reach an acceptable range for the large pore radius can be a positive sign for the strength of the selective treatment method using the ultrashort pulse without the need for intensive increase for the applied pulse.

**Fig. 23:**
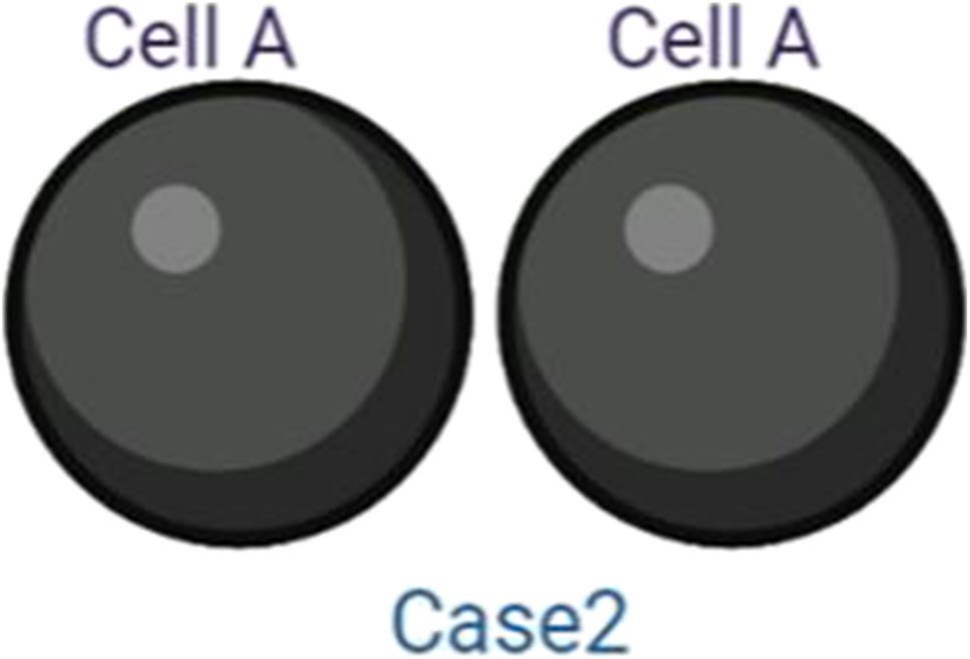
This figure shows the selected case with the need to define the effect of the selective pulse and the effectiveness of the treatment method on the same cell size under the electrofusion. This case is based on the same large cell (two cells A-type)

**Fig. 24:**
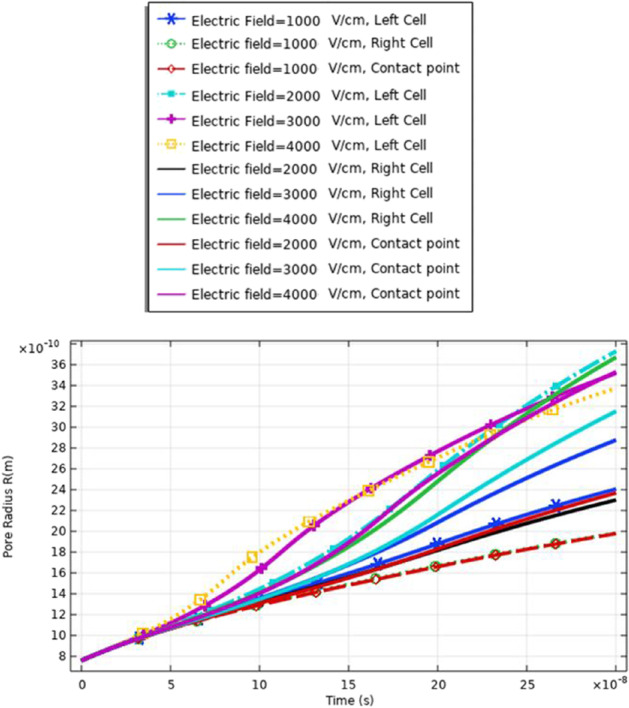
This figure shows the distribution pore radius at different selected points related to the left cell, the right cell, and the contact point between the two cells

**Fig. 25:**
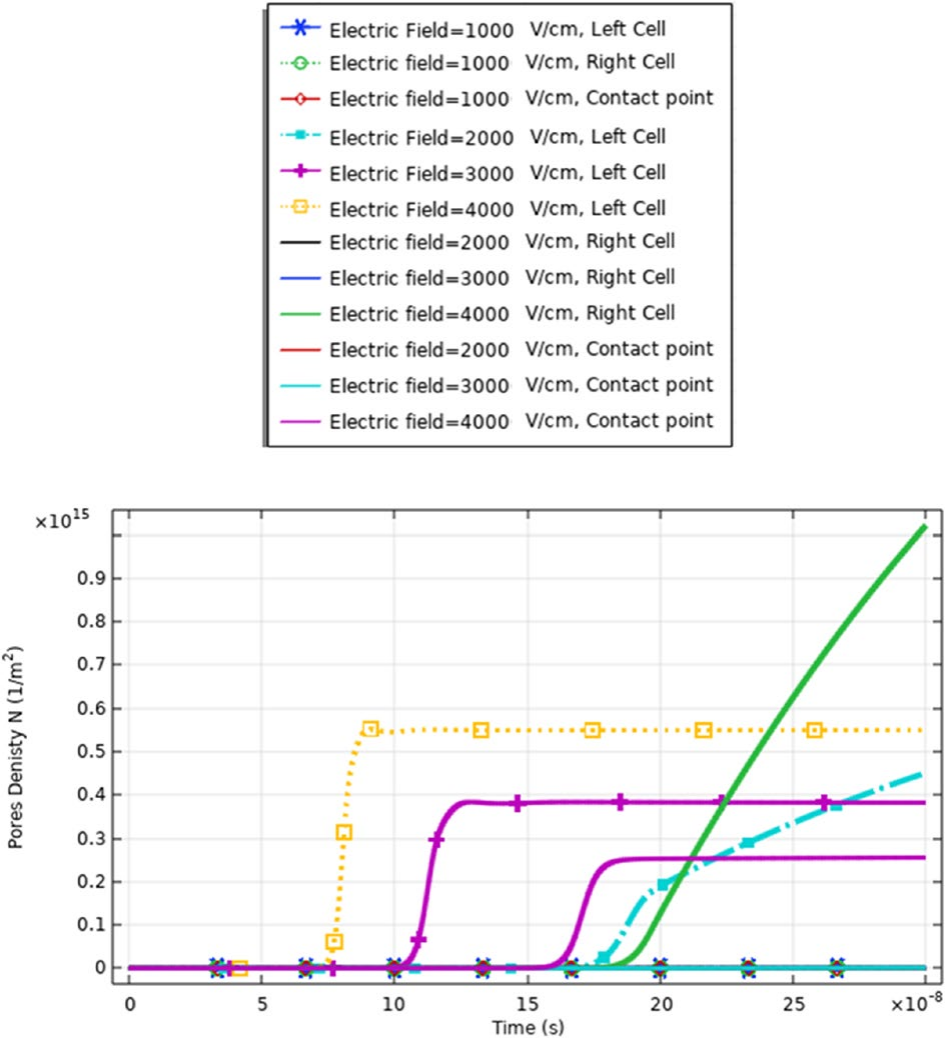
This figure shows the distribution of the density of the pores. The distribution for the same large cell size (two A cells) is described in Table 1 for the dimension of cell A. The presence of the cells for the same type enhances the density of the pores on the right side

**Fig. 26:**
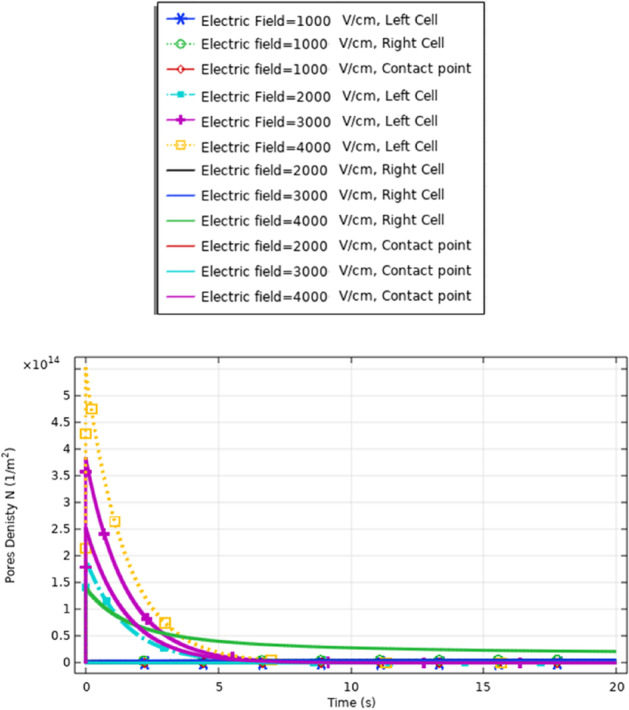
This figure shows the membrane resealing response for the same cell size with the different electric field strength. The represented figure can show the strength of the treatment method to define not only the difference in the electrofusion between the different cell sizes but also the difference in the response for the same cell size

**Fig. 27:**
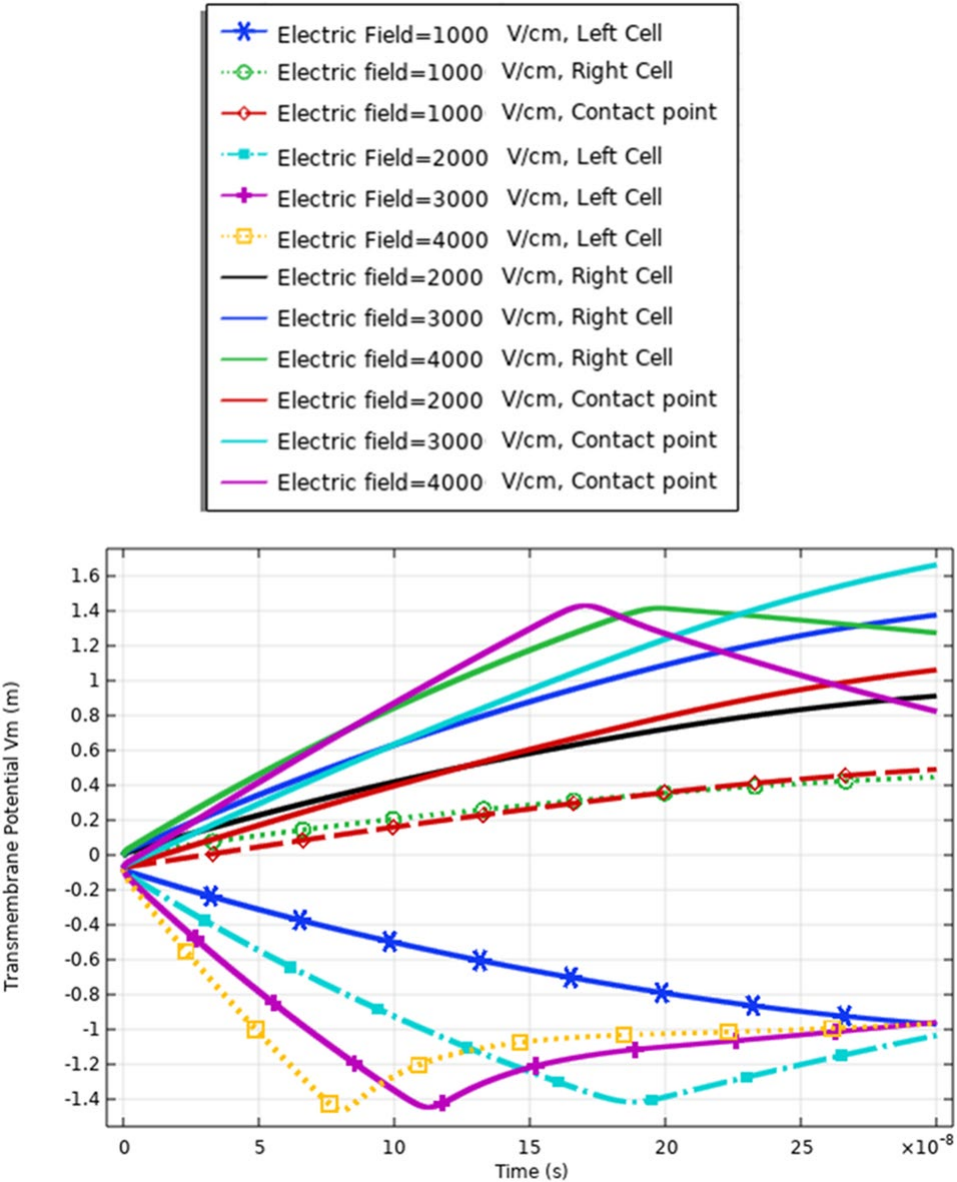
This figure shows the transmembrane potential for the same cell size under the selected treatment method

**Fig. 28:**
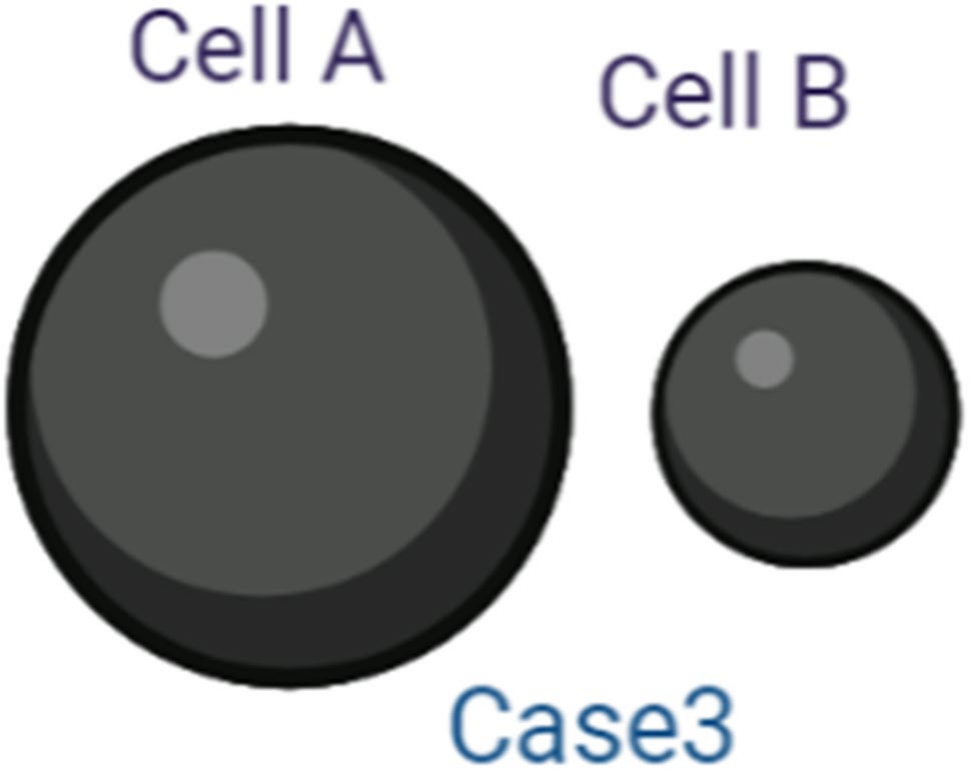
This figure shows the first model case and the distribution of the electric field based on the position of the applied electrodes. The first model to extract the relation between the different two types of cells is based on the change in the diameters as mentioned in Table 1. The effectiveness of the selected pulse differs between the different sizes of the pulse

**Fig. 29:**
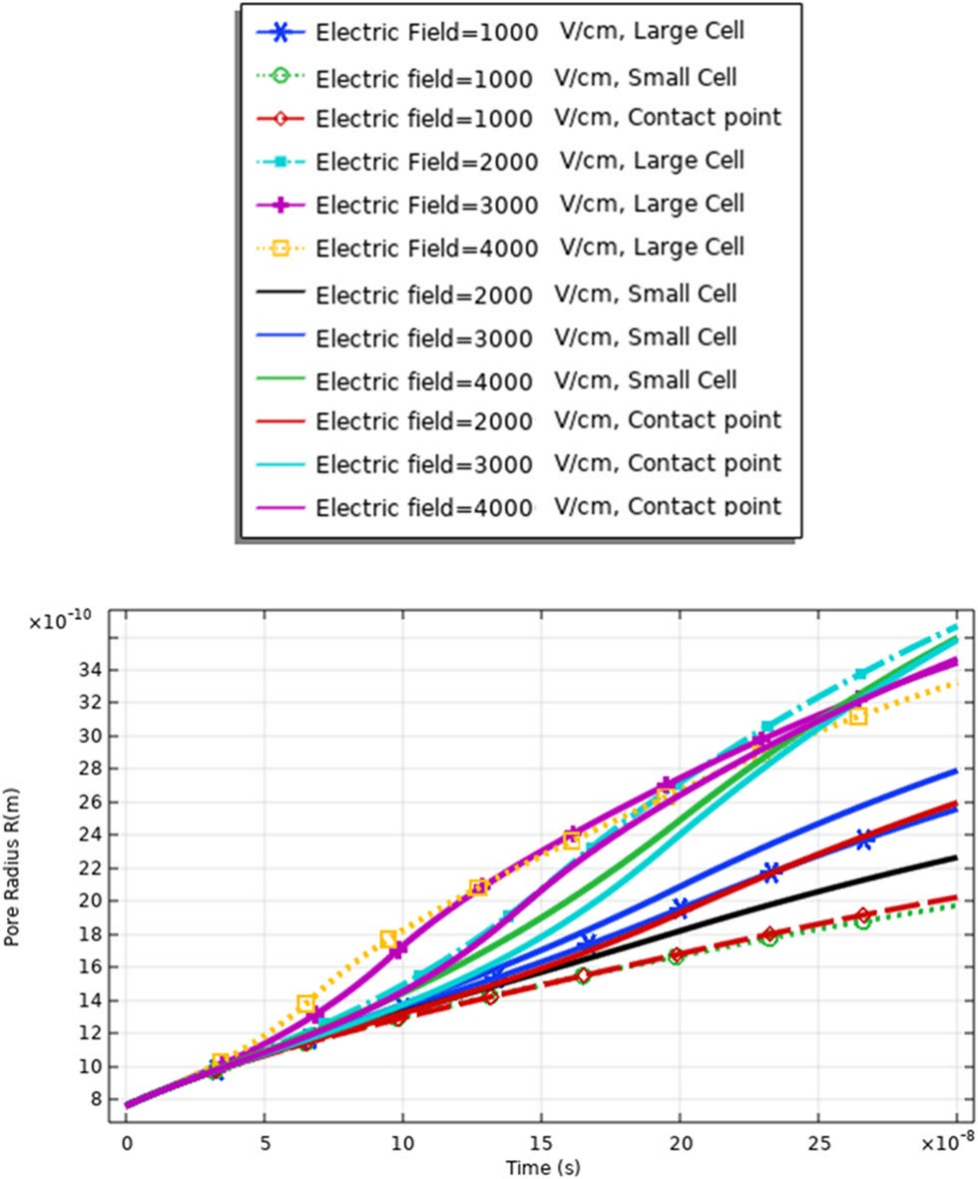
This figure shows the pore radius at different selected points related to the large cell, the small cell, and the contact point between the cells. The response was extracted under the effect of the electric pulse with the nanosecond duration and [1:1:4] kV/cm as a sequence for the electric field

**Fig. 30:**
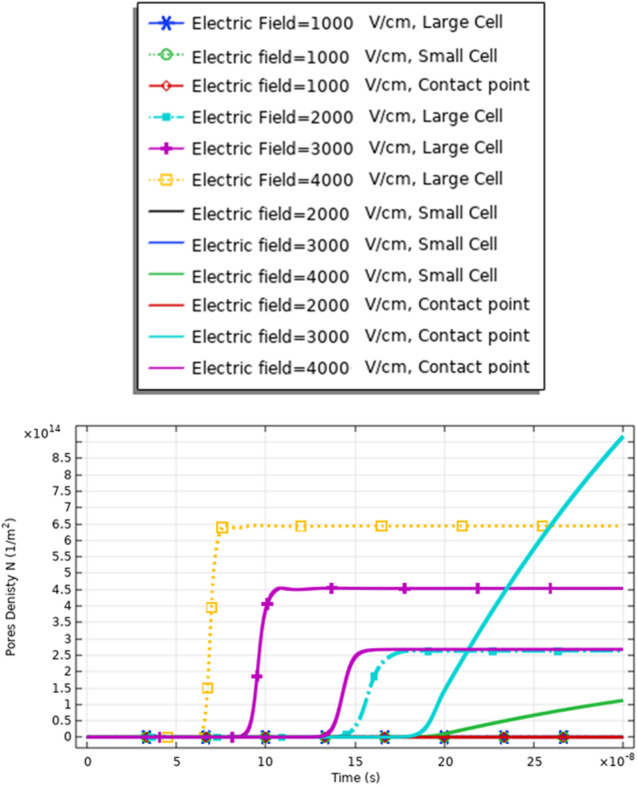
This figure shows the distribution of the density of the pores. The figure data represent the main difference between the different selected points and the main effect of the increment in the electric field on the distribution of the electric field at the small, large, and contact points. The contact point parameter considers assigning for the electrofusion response

**Fig. 31:**
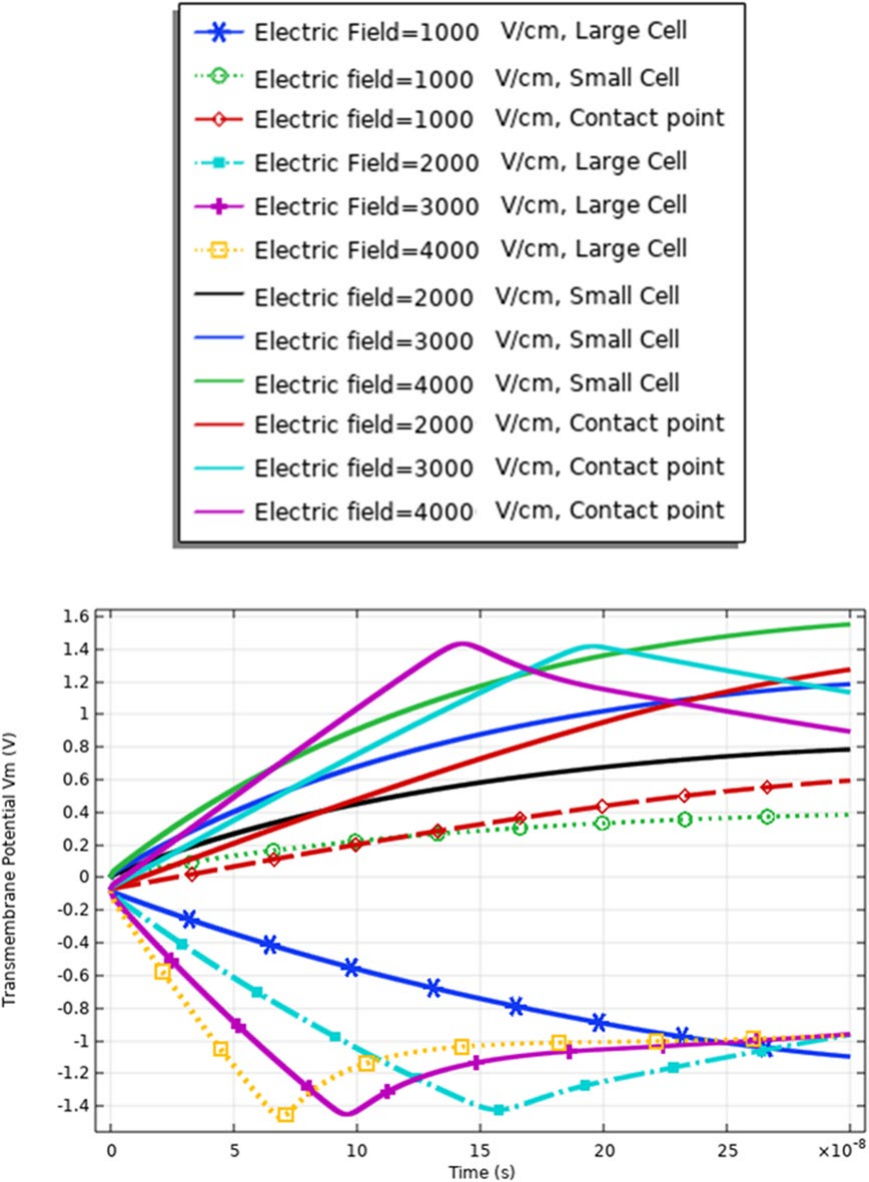
This figure shows the transmembrane potential under the nanosecond electric pulse with the distribution of the electric field [1:1:4] kV/cm. The response for the transmembrane potential is based on the different cells

**Fig. 32:**
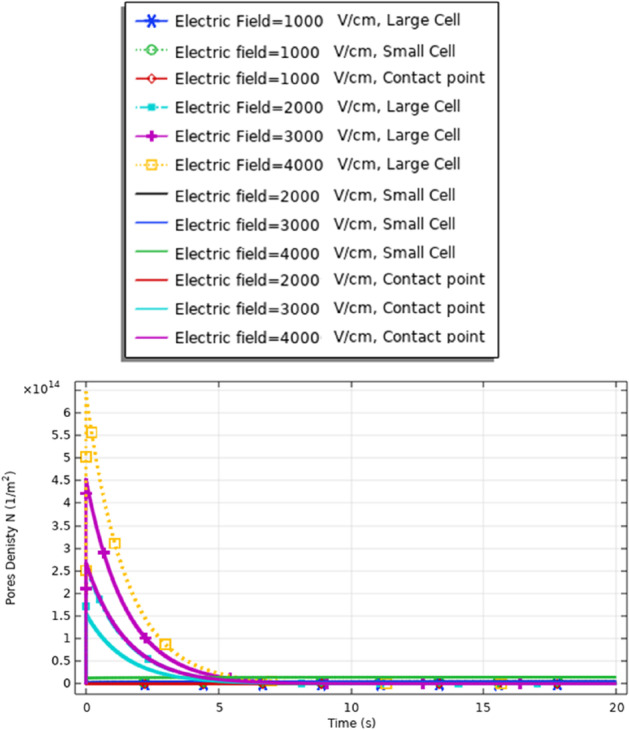
This figure shows the ability of the selected excitation signal to achieve the membrane resealing with the end of the electric pulse

Figure 19 and 22 used to describe the response of the electrofusion for the same cell size for selected CHO cells.

The fluctuation in the formation of the pore radius and the transmembrane potential can be a feature to define the electrofusion that occurs. The delay in the charging time (Fig. 27) for the cells up to reach the critical value of transmembrane potential ($\sim 1 \mathrm{V}$) results from the electrofusion.

Each cell is not only affected by the applied electric field but also affected by the neighbor cells.

The delay time to charge the membrane of the cell under the electrofusion can be obtained by comparing the distribution of the transmembrane potential for the same Cho cells in Fig. 19, same B16-F1 as seen in Fig. 27, and different cells in Fig. 19.

The rapid rise-time pulse allowed penetration not only of the plasma membrane but also of the organelle membrane. The organelle was protected from electroporation by using a slow rise-time pulse.

From Fig. 30, it was found that the density of the pores after 20*${10}^{-8} \mathrm{s}$ equals 9*${10}^{14}{\mathrm{m}}^{-2}$ as a reflection for the interaction between two different cells for the contact point at 3 kV/cm.

From Fig. 21, it was found that the density of the pores after 20*${10}^{-8} \mathrm{s}$ equals 4*${10}^{14}{m}^{-2}$ as a reflection for the interaction between two same small cells for the contact point at 4 kV/cm.

Also, from Fig. 25, it was found that the density of the pores after 20*${10}^{-8}\mathrm{ s}$ equals 0.3*${10}^{15}{\mathrm{m}}^{-2}$ as a reflection for the interaction between two same large cells for the contact point at 4 kV/cm.

A comparison between the membrane resealing analysis in this study, as shown in Figs. 22, 26 and 32, shows that this enhances the viability of the cells compared to cell viability detected in [29].

E. The electrofusion parameters for the same large cells

F. Electrofusion parameters for different cells

## Conclusion and remark

Electrode design and microfluidic system parameters are considered key factors to control electroporation and electrofusion phenomena that have many applications in the medicine and biology sectors.

The performance of the selected system is based on the ability of the excitation signal to combine different advantages, such as ultra-shorted pulse to differentiate between different cell sizes and achieve high-pore density with large-pore radius.

This work provides a high-throughput mathematical model to achieve electroporation and electrofusion with optimal thresholds of the applied electric stimulus pulse. The solutions for the mathematical equations show a high sensitivity of the selected method to differentiate between not only every single cell but also differentiate between the electrofusion between cells of different sizes. In this mathematical analysis, we extracted the ultrashort pulse in the electrofusion that induces contact area between the cells. The definition for the cell membrane penetration can describe by the charging time described in the definition for the transmembrane potential. The success of the electrofusion can described be as the definition for the density of the pores at the contact point between cells and the maximum pore radius can be achieved at a specific time at the contact point between cells.
